# Draft genome sequence of *Marssonina coronaria*, causal agent of apple blotch, and comparisons with the *Marssonina brunnea* and *Marssonina rosae* genomes

**DOI:** 10.1371/journal.pone.0246666

**Published:** 2021-02-05

**Authors:** Qiang Cheng, Junxiang Chen, Lijuan Zhao

**Affiliations:** Key Laboratory of Forest Genetics & Biotechnology of Ministry of Education, Co-Innovation Center for Sustainable Forestry in Southern China, Nanjing Forestry University, Nanjing, China; University of Nebraska-Lincoln, UNITED STATES

## Abstract

*Marssonina coronaria* Ellis & Davis is a filamentous fungus in the class Leotiomycetes that causes apple blotch, an economically important disease of apples worldwide. Here, we sequenced the whole genome of *M*. *coronaria* strain NL1. The genome contained 50.3 Mb with 589 scaffolds and 9,622 protein-coding genes. A phylogenetic analysis using multiple loci and a whole-genome alignment revealed that *M*. *coronaria* is closely related to *Marssonina rosae* and *Marssonina brunnea*. A comparison of the three genomes revealed 90 species-specific carbohydrate-active enzymes, 19 of which showed atypical distributions, and 12 species-specific secondary metabolite biosynthetic gene clusters, two of which have the potential to synthesize products analogous to PR toxin and swainsonine, respectively. We identified 796 genes encoding for small secreted proteins in *Marssonina* spp., many encoding for unknown hypothetical proteins. In addition, we revealed the genetic architecture of the *MAT1-1* and *MAT1-2* mating-type loci of *M*. *coronaria*, as well as 16 tested isolates carrying either *MAT1-1* idiomorph (3) or *MAT1-2* idiomorph (13). Our results showed a series of species-specific carbohydrate-active enzyme, secondary metabolite biosynthetic gene clusters and small-secreted proteins that may be involved in the adaptation of *Marssonina* spp. to their distinct hosts. We also confirmed that *M*. *coronaria* possesses a heterothallic mating system and has outcrossing potential in nature.

## Introduction

The fungus *Marssonina coronaria* Ellis & Davis (Leotiomycetes, Ascomycota) is the causal agent of apple blotch, which is a widespread and devastating disease of apples (*Malus × domestica* Borkh) [1]. This fungus was first reported on wild crabapple in the USA in 1902 [2], and to date, apple blotch has been widely recorded in Asia [3], Europe [4] and both North and South America [5, 6]. In the apple-growing region of China, apple blotch causes 50%–90% defoliation in most orchards during epidemic years [7, 8]. In addition, apple blotch is intractable because the recent increase in the organic farming of apples worldwide requires the limited application of fungicides [9]. Additionally, the emergence of new fungicide-resistant strains in traditional apple-production areas [10] and the lack of stable resistant cultivars [9, 11–14] have led to difficulty in resistance breeding.

*Marssonina coronaria* primarily infects apple leaves, resulting in a blotchy symptom, which is characterized by 3–10 mm diameter dark brown leaf spots. Occasionally, *M*. *coronaria* infections lead to brown depressed spots on fruit surfaces. Severe infections often lead to the chlorosis and defoliation of infested leaves, resulting in reflowering after autumn, which decreases tree vigor and fruit yield [15, 16]. *Marssonina coronaria* invades foliar tissues owing to its hemibiotrophic lifestyle. In the early stage, intercellular hyphae and haustoria develop, and the host cell membrane remains intact. The intracellular hyphae break the host cells’ membranes at approximately 5 days after inoculation, marking the transition to the necrotrophic stage [17]. In addition, the teleomorphic stage (*Diplocarpon mali*) of *M*. *coronaria* may be essential for completing the disease cycle, because the ascospores of the apothecia from overwintered apple leaves are likely to form the primary inoculum [3, 18]. However, the sexual stage of *M*. *coronaria* has rarely been observed, and its mating system is completely unknown.

The fungal genus *Marssonina* comprises approximately 20 species, which are pathogens of many plants, and most have a hemibiotrophic life style [19, 20]. The genomes of *Marssonina brunnea* f. sp. *multigermtubi* (hereafter *M*. *brunnea*) and *Diplocarpon rosae* (anamorph, *Marssonina rosae*) (hereafter *M*. *rosae*), the causal agents of poplar and rose black spot diseases, respectively, have been sequenced [21, 22]. This study aimed to present the genome sequences and annotations of *M*. *coronaria*, identify species-specific carbohydrate-active enzyme (CAZymes), secondary metabolite biosynthetic gene clusters (SM-BGCs) and small-secreted proteins (SSPs) by comparing *Marssonina* spp. genomes, and describe the genetic architecture of mating-type (MAT) loci in *M*. *coronaria*.

## Materials and methods

### Isolation, growth conditions and genomic DNA preparation

**Fig 1:**
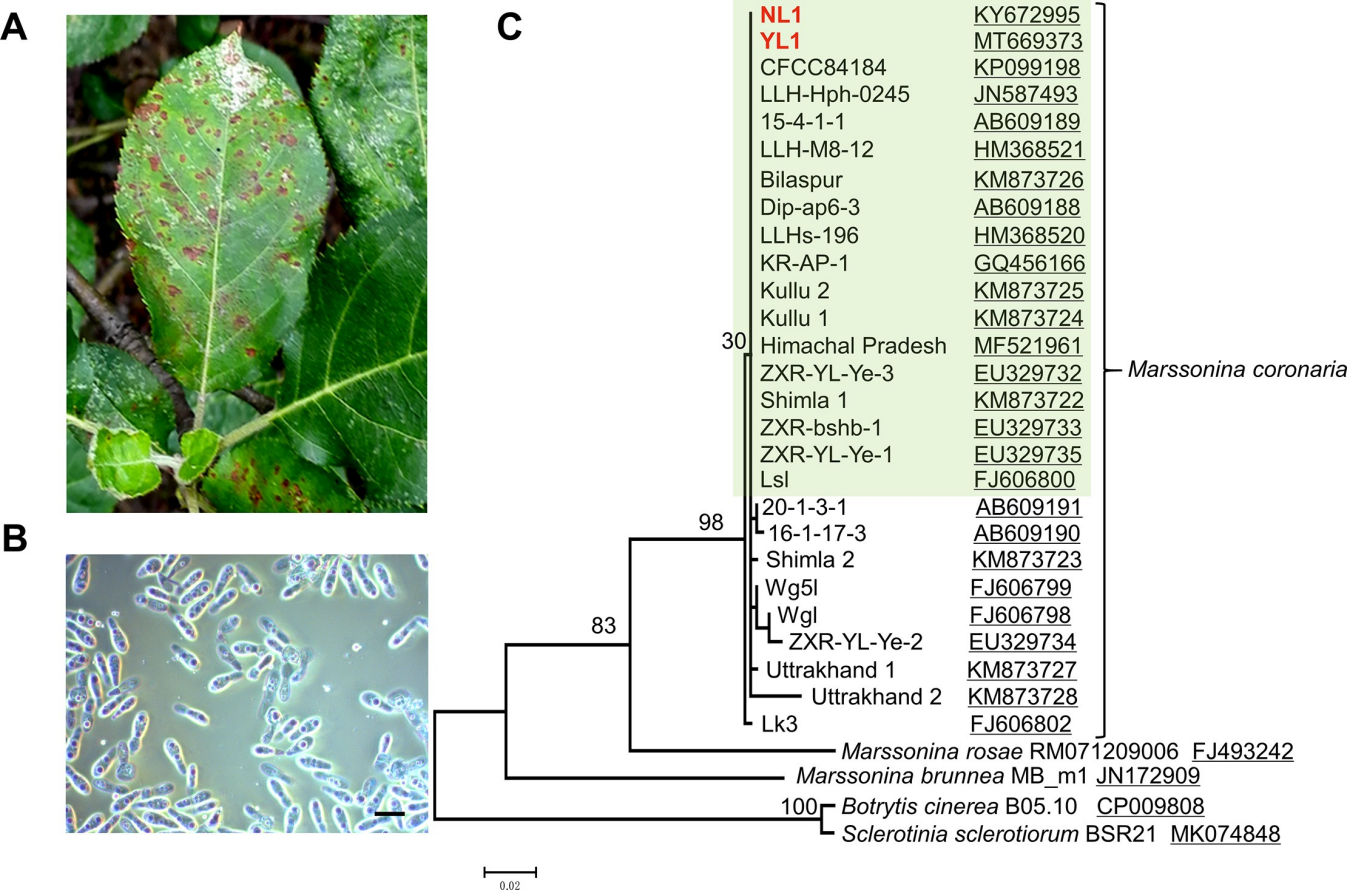
Isolation of *M*. *coronaria* and a phylogenetic analysis using ITS sequences. **(A)** Disease symptoms of apple blotch in the field. **(B)** Conidia of *M*. *coronaria* NL1. Bar, 10 μm. **(C)** Phylogenetic analysis using ITS sequences of *M*. *coronaria*, *M*. *rosae*, *M*. *brunnea*, *B*. *cinerea* and *S*. *sclerotiorum* available in GenBank. The tree was constructed using the maximum likelihood method and tested by 1000 bootstrap replicates. The clade of *B*. *cinerea* and *S*. *sclerotiorum* was selected as an out-group. The *M*. *coronaria* NL1 and YL1 strains are indicated in red. GenBank accession numbers were underlined. Strains/isolates with limited polymorphisms are indicated in light green. The alignment and tree were deposited in Treebase (accession number S27522).

*Marssonina coronaria* was isolated from an apple blotch–infected leaf of a 10-year-old tree (*Malus domestica* Borkh. cv. Red Fuji) in June 2015 at the Nanjing Forestry University campus, Nanjing, Jiangsu, China (Fig 1A). Infected leaves were surface-sterilized with 0.1% mercuric chloride and washed with sterile distilled water. The leaves were cut into approximately 5-mm segments that were placed on potato dextrose agar (PDA) medium at 25°C. After 20 days, colonies with asexual conidia developed on the edge of the leaf disk (Fig 1B). Then, single spores were picked onto an agar plate under a microscope. The DNA of a strain NL1 obtained by single spore isolation was extracted using a DNAsecure Plant Kit (Tiangen, Beijing, China) for genomic sequencing. In addition, 15 strains, YL1–15, of *M*. *coronaria* were isolated using the same method from an apple tree in Yangling, Shaanxi Province, China. The internal transcribed spacer (ITS) regions of strain NL1 and YL1 were amplified by ITS1 and ITS4 primers (S1 Table) [23], sequenced and analyzed by phylogenetic tree (see below).

### Genome sequencing, assembly and annotation

Genomic DNA from *M*. *coronaria* NL1 was sequenced using Illumina HiSeq 2500 platform with 125 bp paired-end reads. The sequencing generated more than 42 million paired-end reads, totaling 6.3 Gb. The raw reads were cleaned by removing the adapter sequences, low-quality sequences (more than 15% bases having a Phred Quality Score under 19), and any reads with more than 5% unknown sequences, designated as “N.” These cleaned reads were assembled using SOAPdenovo v.2.0 [24]. GC content was calculated as the percentage of G and C bases in the total base. RepeatMasker v4.0.5 (http://www.repeatmasker.org) with RMBlastn v2.2.27+ was used to mask the repeats in the genome sequence. Genome annotations were performed using GeneMark-ES with the following parameters:—ES (self‐training),—fungus,—max_intron 3000,—min_gene_prediction 120 [25] and FGENESH with gene models of *M*. *brunnea* [26]. rRNA and tRNA genes were detected using RNAmmer v1.2 [27] and tRNAScan-SEv1.4 [28], respectively. The completeness of the assembled genome was assessed using BUSCO v3 against the eukaryote_odb9 and fungi_odb9 dataset [29]. The draft assembly and annotation of *M*. *coronaria* was deposited in GenBank under the accession number MZNU00000000.1 (BioProject: PRJNA376855; BioSample: SAMN06564146).

### Phylogenetic and polymorphic analyses

Maximum-likelihood trees were constructed for swainsonine synthetases (SwnKs) using MEGA 7.0 [30] with a Jones–Taylor–Thornton model that included all the sites and 1000 bootstrap replicates. The SwnK dataset included the BLASTp hits (E-value = 0 and identity ≥ 50%). A phylogenetic analysis of DNA sequences of ITS and multiple loci were conducted using the maximum-likelihood method with the Tamura–Nei model that included all the sites and 1000 bootstrap replicates. The ITS sequences of isolates/strains of *M*. *coronaria*, *M*. *rosae*, *M*. *brunnea*, *Botrytis cinerea*, and *Sclerotinia sclerotiorum* were obtained from GenBank. The clade of *Botrytis cinerea* and *Sclerotinia sclerotiorum* sequences was selected as an out-group. The DNA sequences of nuclear ribosomal ITS, elongation factor 1-α (*EF1-α*), glyceraldehyde-3-phosphate dehydrogenase (*G3PDH*), heat-shock protein 60 (*HSP60*) and DNA-dependent RNA polymerase subunit II (*RPB2*) were obtained from the genome of *M*. *coronaria* NL1, 13 published genomes of Helotiales fungi and *Blumeria graminis* f. sp. *hordei* DH14 of Erysiphales [21, 22, 31–42] and by homologous cloning from *M*. *coronaria* YL1 (S1 Table). The concatenated DNA sequences of ITS, *EF1-α*, *G3PDH*, *HSP60* and *RPB2* were used to construct the phylogenetic tree. The *B*. *graminis* f. sp. *hordei* DH14 sequences were selected as an out-group for multiple loci phylogenetic analysis. The polymorphic sites and indel sites were analyzed using DNAsp 6 [43].

### Whole-genome synteny comparisons

Whole-genome alignments between the genome of *M*. *coronaria* and those of other Helotiales fungi were performed and visualized using SynMap (CoGe; http://www.genomevolution.org) with BLASTn (E-value ≤ 0.0001) and the quota-align-merge algorithm.

### Identification of CAZymes, secondary metabolite biosynthetic gene clusters and small secreted proteins

The annotated proteins of *M*. *coronaria*, *M*. *brunnea* and *M*. *rosae* were screened for carbohydrate-active modules using the carbohydrate-active enzyme annotation (dbCAN2) [44]. CAZymes that were only identified by DIAMOND or Hotpep tools were further confirmed using the InterProScan web server [45]. Since not all CAzymes were secreted out the cell, the putative secreted CAZymes were further identified by SignalP [46] and SecretomeP [47]. A neural network score of ≥ 0.6 in SecretomeP was used as a threshold. The secondary metabolite biosynthetic gene clusters were identified by antiSMASH [48].

The SSPs were identified on the basis of the following criteria: (1) possessing a typical signal peptide predicted by Signalp5.0 [46]; (2) lacking transmembrane helices in mature proteins predicted by TMHMM [49]; (3) no other subcellular localization (i.e. mitochondria and chloroplast), predicted by TargetP (http://www.cbs.dtu.dk/services/TargetP); and (4) ≤ 250 amino acids in length. The *M*. *rosae* genomic content was duplicated, which led to a duplication of many proteins [22]. Thus, two SSPs with continuous identical amino acid lengths ≥ 15 were screened out as one pair of duplicated proteins. Then these pairs were further confirmed by local alignments of their corresponding genomic DNA sequences with EMBOSS Water (https://www.ebi.ac.uk/Tools/psa/emboss_water/).

### Identification of species-specific CAzymes and SSPs

The species-specific CAZymes and SSPs were identified on the basis of the following criteria: (1) no ortholog in the other two *Marssonina* species was found using the reciprocal best hits (RBH) BLAST method; and (2) the best hits of BLASTp in the other two *Marssonina* species possessed identities < 50%.

### Cloning the MAT1-2 locus and idiomorph-specific PCR

A DNA fragment of *M*. *coronaria MAT1-2-1* was amplified from strain YL7 using one pair of degenerate primers. The flanking sequences of *M*. *coronaria MAT1-2-1* were amplified with primers designed from a *MAT1-2-1* fragment and AP endonuclease (*APN2*) and cytoskeleton assembly control protein (*SLA2*) genes (S1 Table). The PCR products were ligated into the pEASY-Blunt Zero vector (Beijing TransGen Biotech Co., Ltd.) for Sanger sequencing. Idiomorph-specific PCR was conducted with primers designed on the basis of the *M*. *coronaria MAT1-1* and *MAT1-2* idiomorph sequences (S1 Table).

## Results and discussion

### The isolation and identification of *M*. *coronaria* NL1

The strains isolated from lesions of apple leaves (Fig 1A and 1B) were identified by BLAST searching ITS sequences in GenBank. Information showed that NL1 and YL1 had high identities with other reported *M*. *coronaria* stains (98%–100%). A phylogenetic analysis using the ITS sequences of *M*. *coronaria* available in GenBank showed that NL1 and YL1 were confined to the *M*. *coronaria* clade with high bootstrap support (Fig 1C). Of note, the polymorphic sites in the ITS from *M*. *coronaria* were limited. For example, among 18 strains in the main clade of *M*. *coronaria*, only two DNA polymorphic sites and three indels were observed.

### The draft genome of *M*. *coronaria* NL1

*Marssonina coronaria* NL1 was sequenced to generate a draft genome. In total, 50.3 Mbp were assembled into 589 scaffolds having a GC content of 43.96% (Table 1), which were similar to those of *M*. *brunnea* (52 Mb and 42.71%, respectively) [21] and smaller than those of the duplicated *M*. *rosae* genome (66.6 Mb and 47.64%, respectively)[22]. The largest scaffold was 1,297,304 bp, and the N50 value was 231,377 bp. The genome coverage was estimated to be 108.78× by comparing the total sequenced nucleotides to the assembled genome size. The completeness of the *M*. *coronaria* genome was estimated to be 97.7% (296/303) and 99% (287/290) when comparing with single-copy orthologs in the BUSCO eukaryotic and fungal datasets, respectively. In total, 9,355 protein-coding, 136 tRNA and 19 rRNA genes were predicted from a masked genome (masking 143,917 bp simple repeats and 6,869 bp low complexity regions).

**Table 1:** Summary statistics of the *M*. *coronaria* NL1 genome assembly.

Attribute	Value
Estimated genome coverage	108.78
Genome size (bp)	50,267,687
Number of scaffolds	589
GC content (%)	43.96
N50 (bp)^a^	231,377
Largest scaffold (bp)	1,297,304
Busco completeness^b^	97.7% and 99%
Total genes	9,511
Protein-coding genes	9,355
RNA genes	156
Secreted protein genes	620
Small secreted protein genes	187

^a^ N50 indicates the sequence length of the shortest scaffold at 50% of the total genome length.

^b^ The Busco completeness was estimated according to the eukaryote_odb9 and fungi_odb9 dataset, respectively.

### Phylogeny and polymorphism analyses

**Fig 2:**
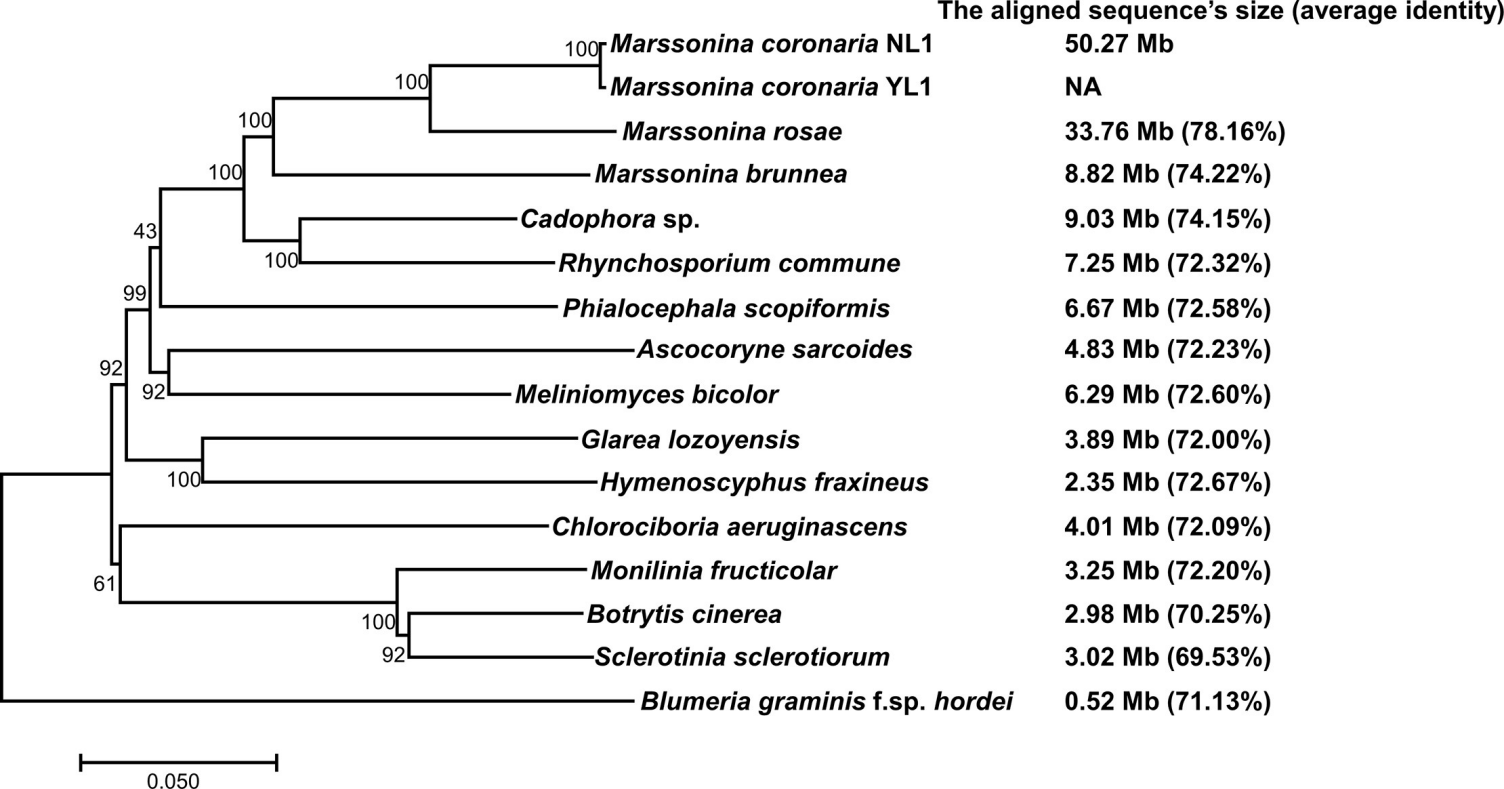
Phylogenetic relationship of 15 fungi in the order Helotiales. The tree was constructed using the maximum-likelihood method based on concatenated DNA sequences of ITS, *EF1-α*, *G3PDH*, *HSP60* and *RPB2*. The inferred phylogenies were tested using 1000 bootstrap replicates. The *B*. *graminis* f. sp. *hordei* DH14 of Erysiphales was selected as an out-group. The length of the genome aligned to the *M*. *coronaria* genome and the average identity of aligned fragments were noted next to each species. The alignment and tree were deposited in Treebase (accession number S27526).

To better understand the evolutionary relationships among species within the order Helotiales, phylogenetic analysis was performed using multiloci DNA sequences (ITS, *EF1-α*, *G3PDH*, *HSP60* and *RPB2*) of 15 Helotiales fungi and *B*. *graminis* f. sp. *hordei* of Erysiphales (S2 Table), and whole-genome alignments between *M*. *coronaria* and its relatives were conducted. As shown in Fig 2, three *Marssonina* species, *M*. *coronaria*, *M*. *rosae* and *M*. *brunnea*, formed a clade with a high bootstrap support, in which *M*. *coronaria* and *M*. *rosae* had the closest relationship with 33.76 Mb of aligned sequences. In contrast, *M*. *coronaria* and *M*. *brunnea* were less closely related, with 8.82 Mb of aligned sequences. *Cadophora* sp. and *Rhynchosporium commune* were clustered with *Marssonina* spp., and 9.03 Mb and 7.25 Mb of the genomic contents, respectively, were aligned to the genome of *M*. *coronaria*. Other Helotiales fungi were in distinct clades and more divergent compared with the *M*. *coronaria* genome (from 6.67 Mb to 2.35 Mb). We also generated *EF1-α*, *G3PDH*, *HSP60* and *RPB2* sequences of YL1 by homologous cloning (Accession No. MT674914–MT674917). In the 8,797-nt sequence of the four protein-encoding genes of NL1 and YL1, 22 DNA polymorphisms and 3 indel polymorphisms were detected, indicating that extensive genetic divergences existed in the two *M*. *coronaria* strains that were from different geographical regions but possessed closely related ITS sequences.

### The species-specific carbohydrate-active enzymes among *Marssonina* spp.

**Fig 3:**
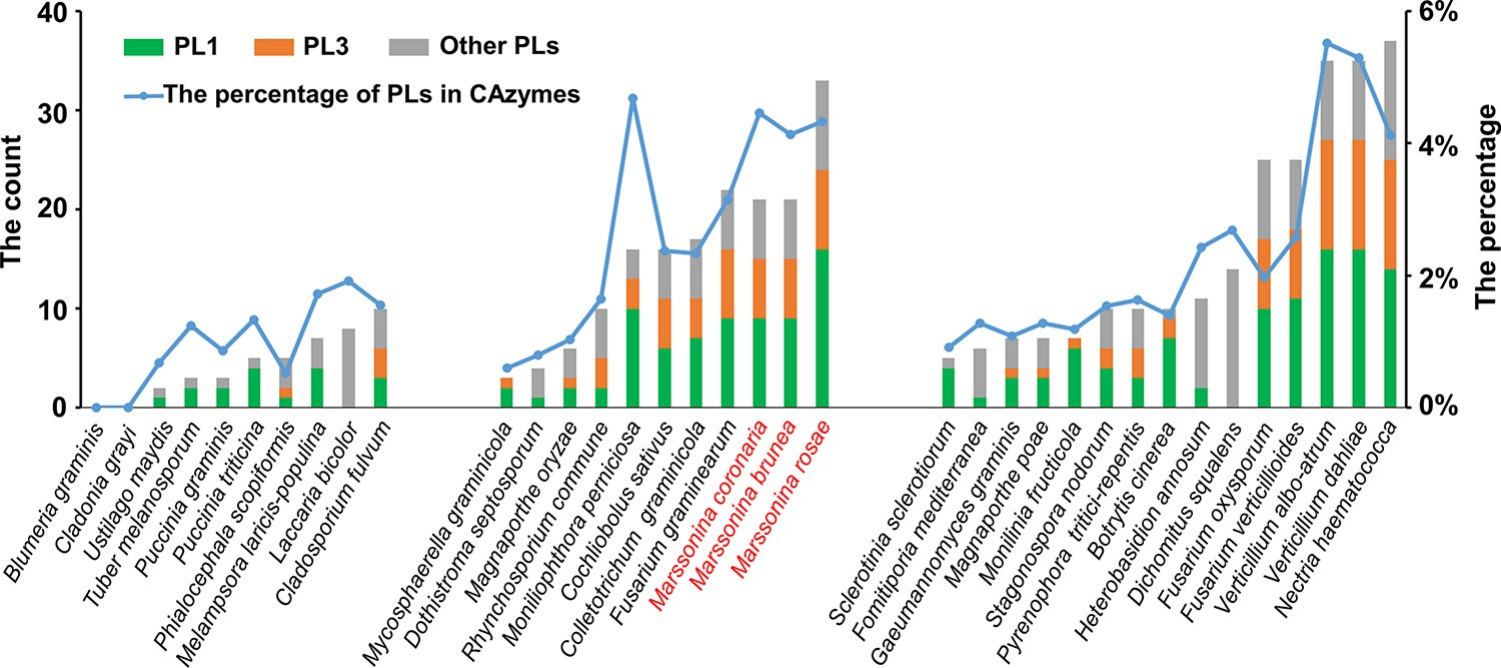
Distribution of Polysaccharide Lyases (PLs) in plant pathogenic fungi. The numbers of PLs (*y*-axis left) from different subfamilies are represented in the stacked bar charts and the percentages of PLs in total CAZymes (*y*-axis right) are represented in the line charts. Other PLs included PL4, -5, -7–12, -14, -15, -17, -20–22, -26, -27, -29, -35 and -36.

To successfully colonize host tissues, phytopathogenic fungi rely on many CAZymes that degrade the polysaccharide barriers of plant cell walls and acquire nutrients [50]. In total, 470, 507 and 762 proteins were identified as CAZymes in *M*. *coronaria*, *M*. *brunnea* and *M*. *rosae*, respectively. A recent comparative survey of multiple fungal genomes revealed that the necrotrophic and hemibiotrophic fungi commonly tend to have more plant cell wall-degrading enzymes than biotrophic fungi [50]. The numbers of CAZymes in the *Marssonina* spp. were greater than in most of the surveyed biotrophic fungi and similar to those of hemibiotrophic fungi (S3 Table). In *M*. *coronaria*, *M*. *brunnea* and *M*. *rosae*, the majority of CAZymes, 61.5% (289/470), 60.4% (306/507) and 61.9% (472/762), respectively, were predicted to function in secretion. Therefore, *Marssonina* spp. have large reservoirs of CAZymes that are secreted into the extracellular space and have the potential to degrade encountered plant cell walls. Compared with a phytopathogenic fungal CAZyme dataset [50], the *Marssonina* spp. possessed higher numbers of polysaccharide lyases (PLs) (Fig 3), which indicated the expansion of pectin lyases and pectate lyases (PL1s) and pectate lyases (PL3s). A similar expansion of PLs was also observed in vascular wilt and root pathogens, such as *Verticillium* spp., *Nectria haematococca* and *Fusarium* spp. (Fig 3) [50], implying a potential requirement of attacking vascular-rich tissues during the infection of *Marssonina* spp.

On the basis of the orthologous analysis using the RBH (no orthologs) method and the BLASTp-based (<50%) identities among *Marssonina* spp., 90 species-specific CAZymes were identified (24 of *M*. *coronaria*, 59 of *M*. *brunnea* and 7 of *M*. *rosae*) (S4 Table). Furthermore, we found that close homologs of 19 species-specific CAZymes were rare in Leotiomycetes, but were common in other taxa, i.e. among the top 10 best BLASTp hits against the NCBI NR database, less than three hits were from Leotiomycetes (Table 2).

**Table 2:** Atypically distributed CAZymes of *M*. *coronaria*, *M*. *brunnea* and *M*. *rosae*.

CAZyme family^a^	Gene name (Gene locus)	Potential substrate^b^	Enzyme activity^b^	Taxa of the top10 best hits^c^
*M*.*coronaria*				
GH16	McADGH16 (B2J93_9182)	Hemicellulose	Xyloglucanase	L 2, D 7, S 1
PL3	McADPL3 (B2J93_5418)	Pectin	Pectate lyase	L 1, D 7, S 2
AA2	McADAA2 (B2J93_2861)	Lignin	Lignin peroxidase	L 2, D 7, S 1
AA3	McADAA3 (B2J93_3628)	Cellulose	glucose 1-oxidase	D 10
		lignin	aryl alcohol oxidase	
AA7	McADAA7 (B2J93_6765)	Cellobiose	glucooligosaccharide oxidase	L 2, D 3, S 5
		chitin/glycoproteins	chitooligosaccharide oxidase	
*M*. *brunnea*				
GH28	MbADGH28 (MBM_02037)	Pectin	Polygalacturonase	L 1, D 8, E 1
GH31	MBM_03122	Hemicellulose	α-xylosidase	P 1, D 6, E 2, S 1
GH43	MbADGH43 (MBM_04126)	Hemicellulose	β-xylosidase	L 1, S 9
		Pectin	α-L-arabinofuranosidase	
GH105	MbADGH105 (MBM_04106)	Pectin	rhamnogalacturonyl hydrolase	L 1, S 4, D 5
CE10	MbADCE10 (MBM_08671)	NA	NA	L 2, E 4, S 3, D 1
CE12	MbADCE12 (MBM_05265)	Pectin	Pectin acetylesterase	L 2,D 8
AA3	MbADAA3 (MBM_08750)	Cellulose	glucose 1-oxidase	D 10
		lignin	aryl alcohol oxidase	
AA7	MbADAA7 (MBM_02730)	Cellobiose	glucooligosaccharide oxidase	L 1, S 8, E 1
		chitin/glycoproteins	chitooligosaccharide oxidase	
AA7	MbADAA7 (MBM_04037)	Cellobiose	glucooligosaccharide oxidase	S 5, Pis 1, B 1, D 1, E 1
		chitin/glycoproteins	chitooligosaccharide oxidase	
AA7	MbADAA7 (MBM_07678)	Cellobiose	glucooligosaccharide oxidase	L 1, S 3, D 3, E 2, Pis 1
		chitin/glycoproteins	chitooligosaccharide oxidase	
AA7	MbADAA7 (MBM_04264)	Cellobiose	glucooligosaccharide oxidase	L 1, S 8, D 1
		chitin/glycoproteins	chitooligosaccharide oxidase	
AA7	MbADAA7 (MBM_03338)	Cellobiose	glucooligosaccharide oxidase	L 2, E 5, D 2, S 1
		chitin/glycoproteins	chitooligosaccharide oxidase	
*D*. *rosae*				
AA3	DrADAA3 (PBP21841)	Cellulose	glucose 1-oxidase	L 1, S 2, D 5, E 2
		lignin	aryl alcohol oxidase	
CBM48	DrADCBM48 (PBP22865)	NA	NA	L 1, D 9

a, The CAZyme family was annotated using the dbcan2 web server.

b, The potential substrates and enzyme activities were annotated in accordance with two references [51, 52].

c, The taxa of the top 10 best hits of BLASTp against the NCBI NR database. The hits from one genus were counted only once. E, Eurotiomycetes; S, Sordariomycetes; L, Leotiomycetes; D, Dothideomycetes; Pis, Pezizomycotina incertae sedis; B, Basidiomycota.

### The secondary metabolism in the *Marssonina* spp.

Phytopathogenic fungi utilize different secondary metabolites as toxins against hosts, mediators for communication, and inhibitors to defeat other competitors. There are four major secondary metabolites in fungi, polyketides, non-ribosomal peptides, cyclic terpenes and tryptophan-derived indole alkaloids, which are synthesized by four central enzymes, polyketide synthase (PKS), non-ribosomal peptide synthase (NRPS), terpene cyclase (TC) and dimethylallyl tryptophane synthase (DMATS), respectively. The genes encoding core synthases and proteins involved in the modification, transportation and regulation of secondary metabolites are often located in single gene clusters on chromosomes, forming a SM-BGC [53, 54].

In total, nine PKS (PKS1–9), three hybrid PKS-NRPS (PKS-NRPS1–3), eight NRPS and eight TC (TC1–8) SM-BGCs were identified in the three *Marssonina* spp. genomes (S5 Table). DMATS clusters were lacking in *Marssonina* spp. *Marssonina coronaria* and *M*. *brunnea* contained two DHN melanin BGCs (PKS2 and PKS7), and *M*. *rosae* contained two pairs owing to a genomic duplication, and they were closely related to the BGCs of *Botrytis cinerea* (BcPKS12 and BcPKS13) [55]. In addition, the BGC of PKS-NRPS2 in *Marssonina* spp. shared two orthologous genes (*fus1* and *fus2*) with the fusarin C BGC of *Fusarium fujikuroi* [56] (S6 Table).

In total, 12 SM-BGCs were species-specific among the *Marssonina* spp., and the core synthases of 5 SM-BGCs (PKS9, PKS-NRPS1, PKS-NRPS3, TC1 and TC5) were also rare in their Leotiomycetes relatives (Table 3). For example, the *M*. *brunnea*-specific TC1 SM-BGC has a high similarity with the PR toxin BGC of *Penicillium chrysogenum* (six orthologous genes with 81%–90% identity levels) (S6 Table) [57]; however, among other Leotiomycetes relatives, only *Hypoxylon* sp. CI-4A had two orthologs that have low identity levels (55%–63%).

**Table 3:** Summary of the species-specific core synthases of the SM-BGCs.

Enzyme	*M*. *coronaria*^a^	*M*. *brunnea*^a^	*M*. *rosae*^a^	Potential product	Taxa of the top10 best hits^b^
PKS8		MBM_04019		NA	L 5, E 2, S 2, D 1
PKS9			PBP25423	NA	L 1, S 4, E 3, D 2
			PBP21839		
PKS-NRPS1			PBP23442	NA	L 1, S 5, E 4
PKS-NRPS3	B2J93_6983			NA	D 4, S 3, X 1, Pis 1, E 1
NRPS2	B2J93_1062			NA	L 5, S 2, E 2, D 1
NRPS3	B2J93_4402			NA	L 4, D 3, X 1, C 1, E 1
NRPS5	B2J93_1626			NA	L 8, D 2
NRPS8		MBM_06951		NA	L 7, D 2, E 1
Tc1		MBM_07677		PR toxin	L 1, S 4, D 4, E 1
Tc4		MBM_04258		NA	L 2, S 4, D 2, E 2
Tc5		MBM_08380		NA	L 5, S 2, E 2, D 1
Tc7	B2J93_6506			NA	L 3, B 6, D 1

a, Gene loci of core synthases.

b, The taxa of the top 10 best BLASTp hits against the NCBI NR database. The hits from one genus were counted only once. E, Eurotiomycetes; S, Sordariomycetes; L, Leotiomycetes; D, Dothideomycetes; X, Xylonomycetes; Pis, Pezizomycotina incertae sedis; C, Lecanoromycetes; B, Basidiomycota.

**Fig 4:**
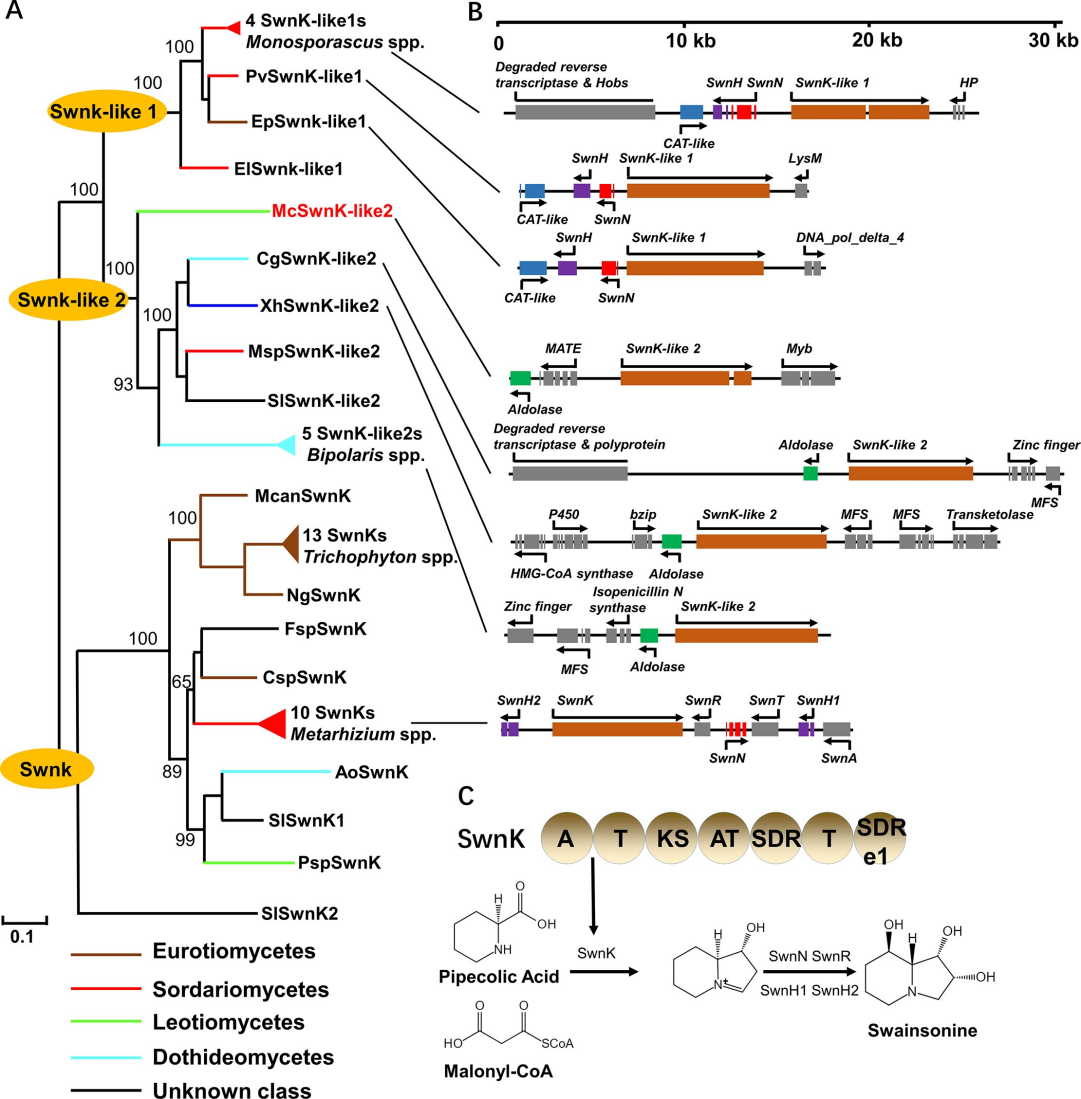
The swainsonine BGCs and their close homologs. **(A)** Phylogenetic analysis of the core synthases SwnK and SwnK-like proteins. The tree was constructed using the maximum-likelihood method, and the inferred phylogenies were tested using 1000 bootstrap replicates. The branches of different taxonomic classes are represented by distinct colors. The alignment and tree were deposited in Treebase (accession number S27543). **(B)** Organization of the swainsonine and homologous BGCs. Boxes represent the coding regions of the predicted genes interrupted by introns. Arrows indicate the orientations of the coding sequences. **(C)** Predicted functions of SwnKs.

A BLAST search against the NCBI NR database revealed that the *M*. *coronaria*-specific PKS-NRPS3 was closely related to SwnK. Swainsonine is a neurotoxic alkaloid produced by several animal and plant pathogenic fungi [58]. A phylogenetic analysis revealed that the homologs of PKS-NRPS3 have a patchy distribution, in which fungal proteins from distinct taxa constituted highly supported clades (Fig 4A, S7 Table). One clade included the SwnK of *Metarhizium robertsii* that were required for swainsonine biosynthesis and the SwnKs from 11 swainsonine-producing fungi [58]. In contrast, another clade containing two subclades (SwnK-like1 and -like2) did not have any member supported by experimental evidence. The *M*. *coronaria*-specific PKS-NRPS3 belonged to the SwnK-like2 subclade. There were seven swainsonine BGCs in the *Metarhizium* spp., *SwnK*, *SwnH1*, *SwnH2*, *SwnN*, *SwnR*, *SwnT* and *SwnA*. *SwnN* and *SwnH* also existed in the flanking region of SwnK-like1, but no synteny was observed between the flanking region of SwnK-like2 and SwnK (Fig 4B). SwnK, SwnK-like1 and SwnK-like2 share the same catalytic domain architecture, including adenylylation (A), phosphopantetheine-binding/thiolation (T), b-ketoacyl synthase (KS), acyltransferase (AT), reductase (SDR), and thioester reductase (SDR e1) domains (Fig 4C). SwnK catalyzed pipecolic acid and malonyl-CoA to form a heterocyclic intermediate of swainsonine [59]. Therefore, SwnK-like1 and -like2 have the potential to mediate reactions similar to those of SwnK that are involved in the synthesis of analogous derivatives of indolizidine alkaloids.

### Amount of small secreted proteins of *Marssonina* spp. were novel proteins

*Marssonina coronaria*, *M*. *brunnea* and *M*. *rosae* are hemibiotrophic pathogens, which feed on living plant cells and maintain host cell viability during the early infection stages [17, 60, 61]. Hemibiotrophs rely on effectors to suppress the plant immune system and reprogram the infected tissue [62]. In accordance with the features of known effectors, candidates should be small, secreted proteins (SSPs), and many show no obvious homology to known proteins [63]. We previously reported a large expansion of the SSPs of LysM effectors (24 members) and IGY proteins (107 members) in *M*. *brunnea* [61, 64]. However, using a recursive BLAST search, we found no such expansion of LysM SSPs, and no IGY motifs in *M*. *coronaria* and *M*. *rosae* annotated proteins.

**Fig 5:**
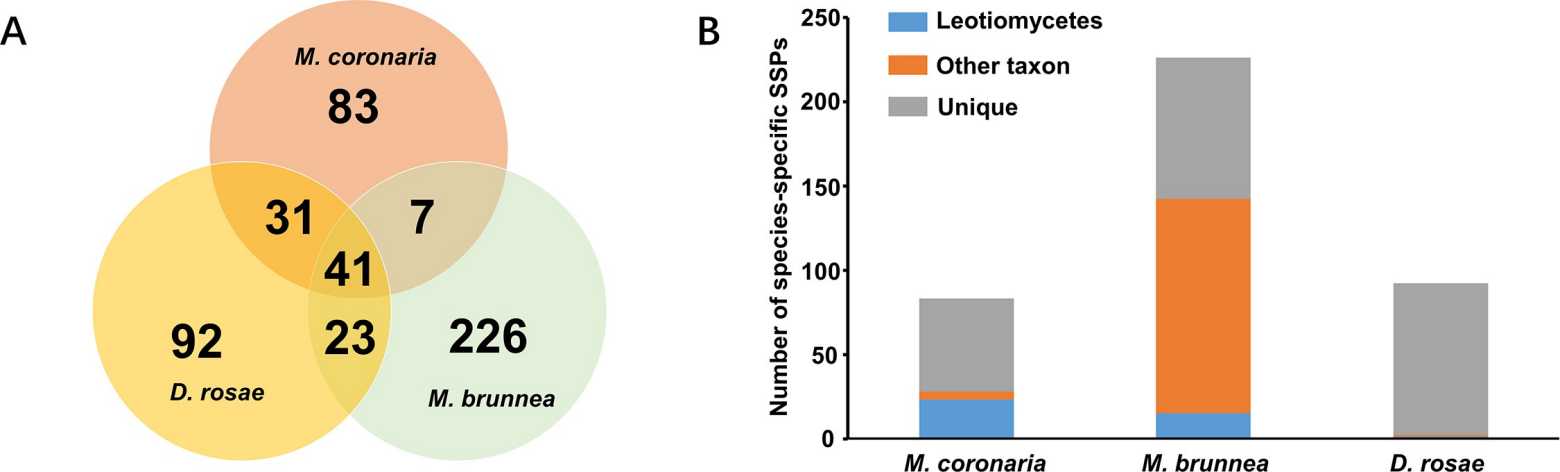
The small secreted proteins of *Marssonina* spp. **(A)** Venn diagram displaying species-specific and shared SSPs in three *Marssonina* spp. The shared sets were orthologs confirmed using the RBH BLAST method, and the species-specific SSPs were SSPs without orthologs, which was confirmed using the RBH BLAST method, and lacking highly identical homologs (≥ 50% identity). **(B)** The taxa distribution of the best hits of species-specific SSPs. The analysis was based on BLASTp searches against the NCBI NR database.

In total, 6.63% (620/9355), 6.73% (927/13761) and 8% (802/10027) proteins of *M*. *coronaria*, *M*. *rosae* and *M*. *brunnea* proteomes were predicted as secreted proteins, in which 187, 285 and 324 proteins with less than or equal to 250 amino acids were considered to be SSPs. More half of the SSPs (50.3%, 65.3% and 51.5% in *M*. *coronaria*, *M*. *rosae* and *M*. *brunnea*) were cysteine-rich proteins (≥ 4 cysteine residues). *M*. *rosae* contained 58 SSP pairs owing to a genomic duplication. There were 41 common SSP orthologs shared in the three *Marssonina* spp., while 83, 92 and 226 SSPs of *M*. *coronaria*, *M*. *rosae* and *M*. *brunnea*, respectively, had no orthologs and no homologs with ≥ 50% identities in the other two relatives. These were referred to as species-specific SSPs (Fig 5A; S8–S10 Tables). Furthermore, a BLASTp search against the NCBI NR database revealed that amount of species-specific SSPs were unique in the NR database (55 of 83 in *M*. *coronaria*, 90 of 92 in *M*. *rosae* and 84 of 226 in *M*. *brunnea*). The best hits of more than half of the *M*. *brunnea*-specific SSPs (127 of 226) belonged to taxa other than Leotiomycetes, while, in contrast, the best hits of most *M*. *coronaria-* and *M*. *rosae-*specific SSPs were in Leotiomycetes relatives (Fig 5B; S8–S10 Tables).

### Mating system

In the Ascomycota fungi, most sexual reproduction is controlled by a single genetic locus, the *MAT* locus, which has alternative forms (idiomorphs) with highly divergent sequences, *MAT1-1* and *MAT1-2*. *MAT1-1* is characterized by the *MAT1-1-1* gene that encodes an alpha-box protein, and *MAT1-2* typically carries the *MAT1-2-1* gene encoding a high mobility group (HMG) motif-containing protein. Both *MAT1-1* and *MAT1-2* are generally flanked by the *APN2* and *SLA2* genes. Strains of heterothallic fungi containing one locus can mate with strains carrying the opposite locus. In contrast, homothallic fungi often contain both *MAT1-1-1* and *MAT1-2-1* genes within a single strain, which enables self-crossing [65].

**Fig 6:**
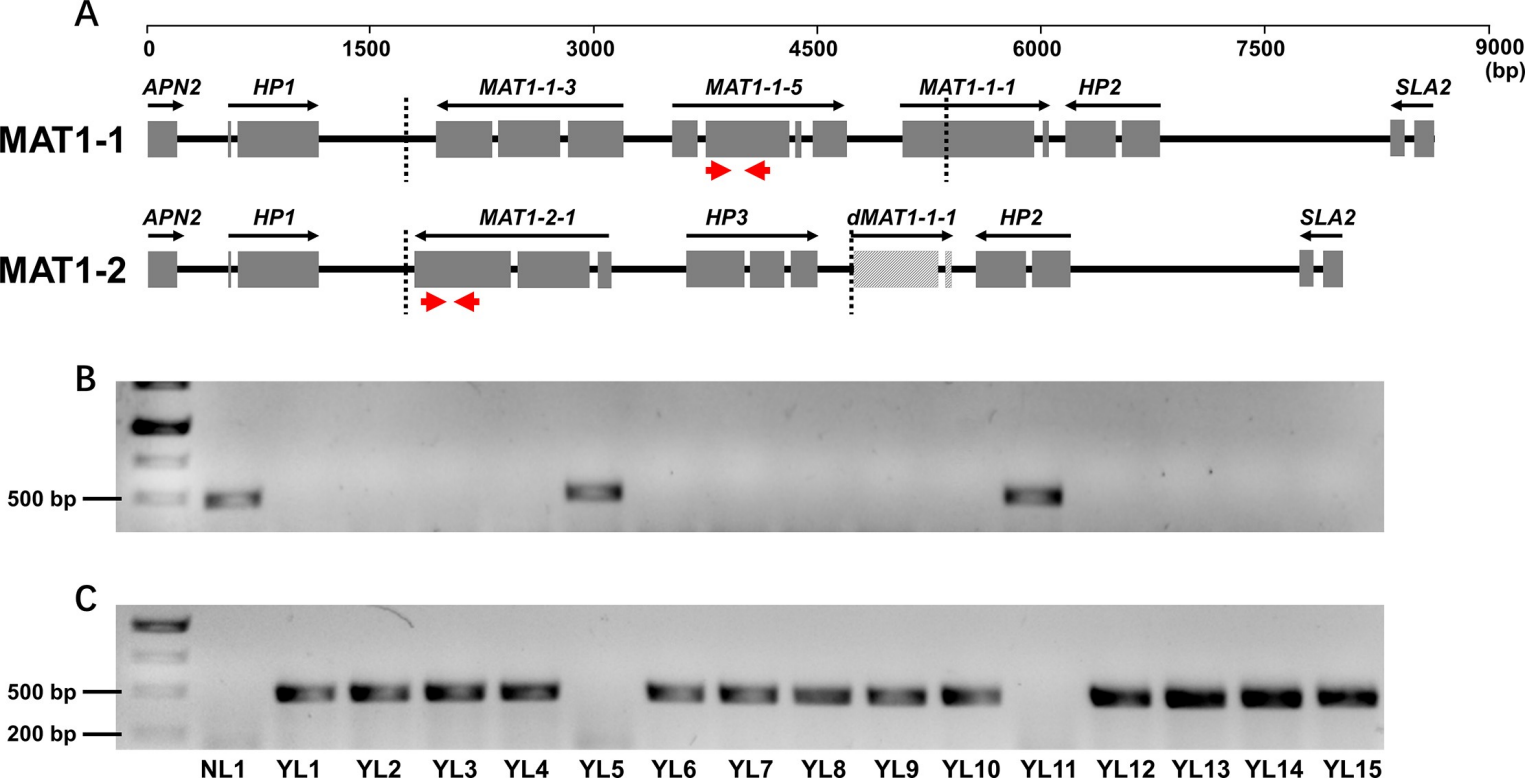
The mating-type loci and detection of mating types of *M*. *coronaria*. **(A)** Structures of the *MAT1-1* and *MAT1-2* loci. Solid boxes represent the coding regions of the predicted genes interrupted by introns. Black arrows indicate the orientations of the coding sequences. Dotted lines mark the sizes of the unique sequences of the idiomorphs. Red arrows indicate idiomorph-specific primers. **(B,C)** Detection of *MAT1-1*, product size 468 bp (B) and *MAT1-2*, product size 476 bp (C) with idiomorph-specific primers. M, DNA ladder; lanes 1–16, sixteen *M*. *coronaria* isolates, NL1 and YL1–15.

The genome of *M*. *coronaria* NL1 possesses a single *MAT1-1* locus between *APN2* and *SLA2* (Accession No. MT819950) (Fig 6A). Five genes were predicted from this region, *MAT1-1-1*, *MAT1-1-3*, *MAT1-1-5* and two hypothetical protein genes (*HP1* and *HP2*). In addition to *HP1* and *HP2*, the architecture of the *MAT1-1* locus of *M*. *coronaria* is identical to that of the closely related *R*. *commune* [32]. *HP1* and *HP2* are completely unique to *M*. *coronaria* and lack homologs (E-value ≤ 10) in the NR database of NCBI. The long-range amplification with primers designed to the flanking *APN2* and *SLA2* genes revealed the genetic structure of the *MAT1-2* locus (Accession No. MT819951) in the isolate YL7 (Fig 6A). *MAT1-2-1*, another hypothetical protein gene (*HP3*), truncated MAT1-1-1 (679 bp, 99% identity), and nearly identical *HP1* and *HP2* genes were predicted in this region of YL1. *HP3* had homologs in *M*. *brunnea* and *Rhynchosporium agropyri* that were also proximal to *MAT1-2-1*. Truncated *MAT1-1-1* fragments were detected in the *MAT1-1* locus of the Helotiales fungi *B*. *cinerea* [66], *R*. *agropyri* [32] and *Monilinia* spp. [67], and they were presumed to be the products of evolution from the homothallic *MAT1* locus to heterothallic locus through multiple recombination and deletion events. A comparison of the two *MAT1* loci of *M*. *coronaria* revealed that the sizes of the idiomorphs were 3,618 bp (*MAT1-1*) and 2,955 bp (*MAT1-2*). Amplification with idiomorph-specific primers revealed that single isolates only carry one of the two opposite idiomorphs (Fig 6B and 6C), implying a heterothallic system in *M*. *coronaria*.

## Conclusions

*Marssonina brunnea*, *M*. *rosae* and *M*. *coronaria* are three of the most widespread and destructive phytopathogens in *Marssonina*. The genomes of the first two fungi have been reported, and here, we provide the genome sequence of *M*. *coronaria*. A comparison of the three *Marssonina* genomes revealed species-specific proteins, some of which had either atypical (19 CAZymes, proteins in 5 SM-BGCs), or unique (229 SSPs) distributions. These phenomena likely resulted from dynamic gene duplication and loss, horizontal gene transfer or strong diverse selection. These evolutionary forces are often closely related to environmental adaptation. Therefore, the species-specific proteins discovered in this study may serve as keys to understanding the specific interactions between *Marssonina* spp. and their hosts, as well as their adaptation in distinct ecological niches.

Outcrossing pathogens may have higher evolutionary potential to overcome plant resistance strategies than asexual pathogens. Here, we revealed the unique architecture of the *MAT1* locus of *M*. *coronaria*, in which two *M*. *coronaria*-specific hypothetical protein genes (*HP1* and *HP2*) flanked the idiomorphs. We also confirmed the heterothallic system in isolates from Yangling and Nanjing City, China that exclusively carry either the *MAT1-1* or *MAT1-2* locus. Therefore, *M*. *coronaria* possesses the genetic potential to outcross, which may lead to altered pathogenicity through the recombination of virulence-related genes.

## Supporting information

S1 Raw images Whole gel photos. (A) Whole gel photo for Fig 6B. (B) Whole gel photo for Fig 6C. The gels were photographed by GelDoc XR (Bio-Rad, Germany).
(PDF)

S1 Table Primers used for gene cloning and idiomorph-specific PCR. (DOCX)

S2 Table The phylogenetic sequence of 15 Helotiales fungi and *Blumeria graminis* f. sp. *hordei* DH14. (DOCX)

S3 Table The summary of CAZymes of thirty-six phytopathogenic fungi. (DOCX)

S4 Table Species-specific CAZymes of *M*. *coronaria*, *M*. *brunnea* and *M*. *rosae*. (DOCX)

S5 Table The summary of core synthases of secondary metabolism in *Marssonina* spp. (DOCX)

S6 Table DHN melanin, Fusarin and PR toxin BGCs in *Marssonina* spp. (DOCX)

S7 Table The summary of homologs of PKS-NRPS3 of *M*. *coronaria* used in phylogenetic analysis. (DOCX)

S8 Table The summary of the small secreted proteins of *Marssonina coronaria*. (DOCX)

S9 Table The summary of the small secreted proteins of *Marssonina brunnea*. (DOCX)

S10 Table The summary of the small secreted proteins of *Marssonina rosae*. (DOCX)
