# Supplementary figures and images for: Draft genome sequence of Marssonina coronaria, causal agent of apple blotch, and comparisons with the Marssonina brunnea and Marssonina rosae genomes
Source: PLoS One. 2021 Feb 5;16(2):e0246666. doi: 10.1371/journal.pone.0246666 (PMC7864672; doi:10.1371/journal.pone.0246666)

A

4500 bp  
3000 bp  
2000 bp  
1200 bp  
800 bp  
500 bp  
200 bp

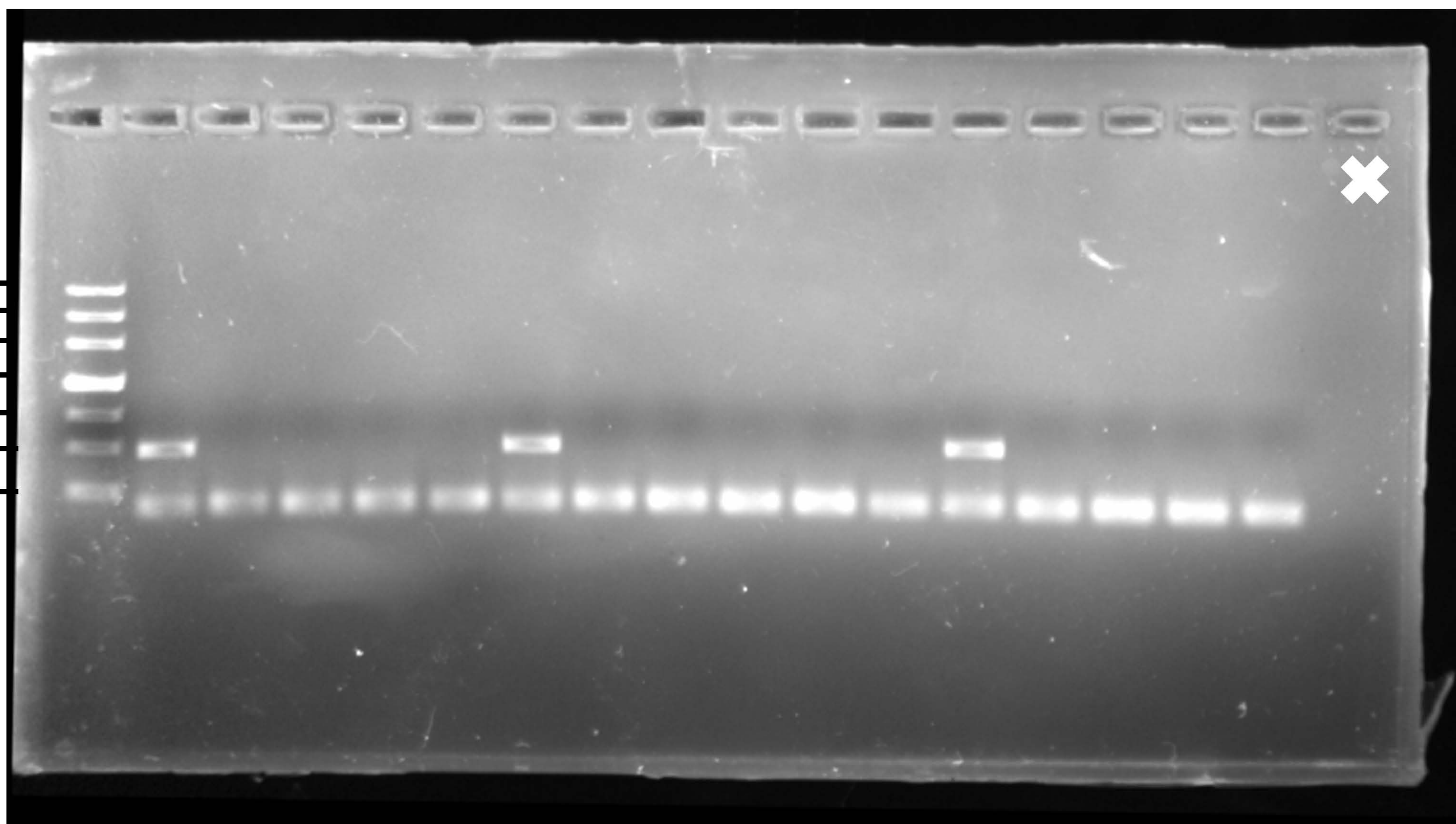

B

4500 bp  
3000 bp  
2000 bp  
1200 bp  
800 bp  
500 bp  
200 bp

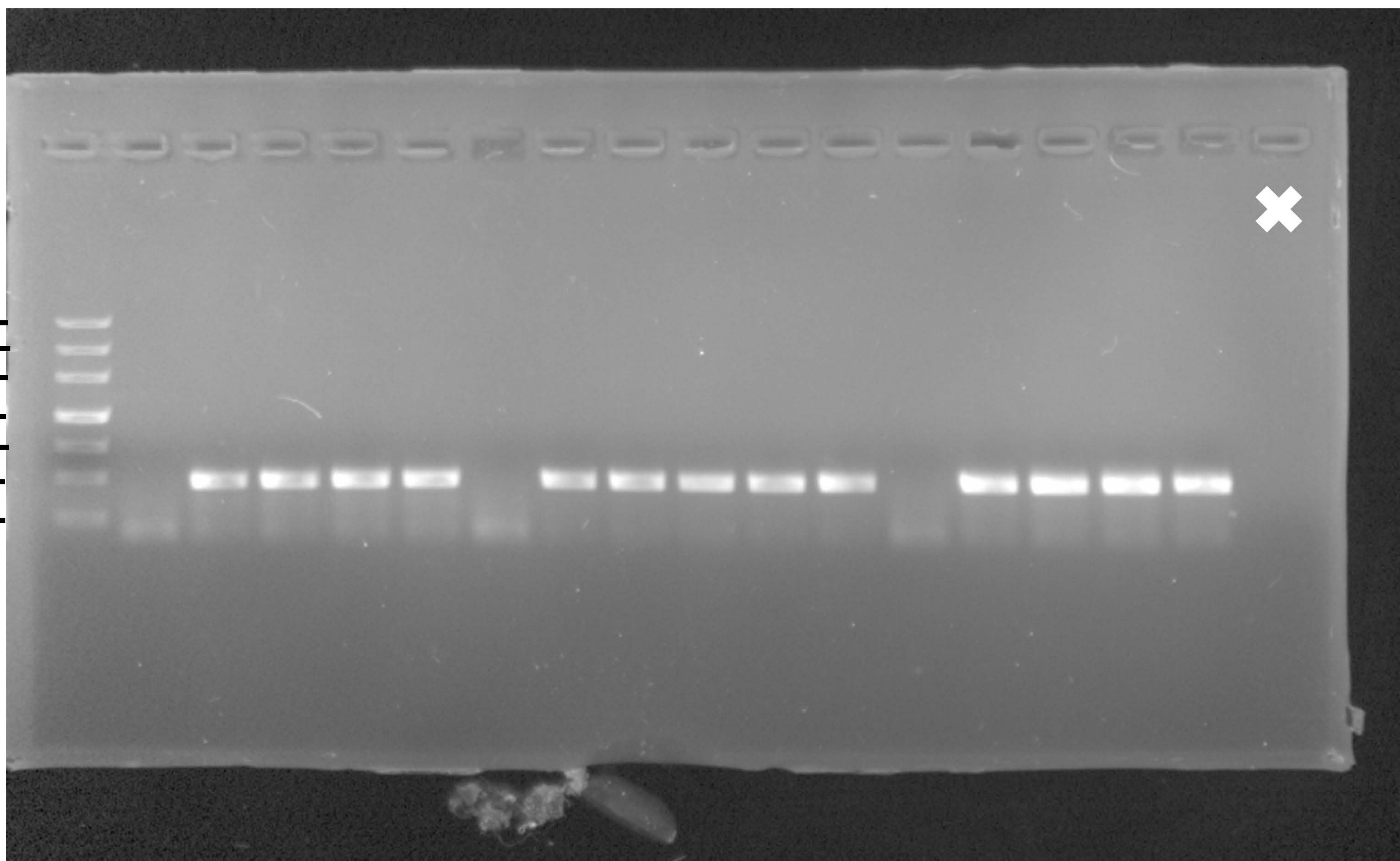

Supplement: S1 Raw images — (A) Whole gel photo for Fig 6B. (B) Whole gel photo for Fig 6C. The gels were photographed by GelDoc XR (Bio-Rad, Germany). (PDF) [file pone.0246666.s001.pdf]
