# Supplementary material for: Draft genome sequence of Marssonina coronaria, causal agent of apple blotch, and comparisons with the Marssonina brunnea and Marssonina rosae genomes
Source: PLoS One. 2021 Feb 5;16(2):e0246666. doi: 10.1371/journal.pone.0246666 (PMC7864672; doi:10.1371/journal.pone.0246666)
Supplement: S1 Table — (DOCX) [file pone.0246666.s002.docx]

**S1 Table.** Primers used for gene cloning and idiomorph-specific PCR

| Primer name | sequence | note |
| --- | --- | --- |
| ITS | 5’-TCCGTAGGTGAACCTGCGG-3’  5’-TCCTCCGCTTATTGATATGC-3’ | Amplify phylogenetic DNA sequences *M. coronaria* YL1 |
| EF1-α | 5’-ATGCACATCAACGTGGTCGTTATC-3’  5’-TTATTTGCCCTTGGTAGCCTTGAC-3’ | Amplify phylogenetic DNA sequences *M. coronaria* YL1 |
| G3PDH | 5’-ATGGCTCCTACAAAGGTTGGCATC-3’  5’-CTACGCTCCATCAACCTTCGCAAT-3’ | Amplify phylogenetic DNA sequences *M. coronaria* YL1 |
| HSP60 | 5’-ATGCAGCGAGCATTGACAACAACA-3’  5’-TTACATCATTCCGCCCATGCCGCC-3’ | Amplify phylogenetic DNA sequences *M. coronaria* YL1 |
| RPB2 | 5’-ATGGCGGACACATACGAAGATGGC-3’  5’-CTACGCGATTGGAGTCATGAGTCC-3’ | Amplify phylogenetic DNA sequences *M. coronaria* YL1 |
| MAT1-2-1-Degenerate | 5’-CGAGACAACAAGATGCCTCGNGTNTTY-3’  5’-TCTTCTTGGTCATCCGACGYTTYTTYTC-3’ | Based on two conserved amino acid block (FLRDNKMPRVF/ EKKRRMT of MAT1-2-1 of *Marssonina brunnea*, *Rhynchosporium secalis* and *Cadophora* sp. DSE1049.  Degenerate primers for a 674 bp fragment of M. coronaria MAT1-2-1 |
| MAT1-2-1-APN2 | 5’-GAAGAAGGAAAAGAACACCGAATG-3’  5’-GCAACTGATGTCAACAATCGTGA-3’ | Amplify flanking sequence of M. coronaria MAT1-2-1 (APN2 direction) |
| MAT1-2-1-SLA2 | 5’-GTAAAGTAGGGCATGCTCTTCCT-3’  5’-AGATACTACAGCTGGAGAACAACC-3’ | Amplify flanking sequence of M. coronaria MAT1-2-1 (SLA2 direction) |
| MAT1-1-idiomorph-specific | 5’-ATTCGCAGTCTCATACGCTACAT-3’  5’-AGCCTTTGGAATCTTCTGCTGTA-3’ | Idiomorph specific primers of *M. coronaria* MAT1-1 |
| MAT1-2-idiomorph-specific | 5’-GAAGTTGAGTGGTCTATGAGCCA-3’  5’-CCCTACTTTACCCAGACTATGCC-3’ | Idiomorph specific primers of *M. coronaria* MAT1-2 |
