# Supplementary material for: Draft genome sequence of Marssonina coronaria, causal agent of apple blotch, and comparisons with the Marssonina brunnea and Marssonina rosae genomes
Source: PLoS One. 2021 Feb 5;16(2):e0246666. doi: 10.1371/journal.pone.0246666 (PMC7864672; doi:10.1371/journal.pone.0246666)
Supplement: S2 Table — (DOCX) [file pone.0246666.s003.docx]

**S2 Table.** The phylogenetic sequence of 15 Helotiales fungi and *Blumeria graminis* f. sp. *hordei* DH14

|  | Species | ITS | RPB2 | EF1a | HSP60 | G3PDH |
| --- | --- | --- | --- | --- | --- | --- |
| 1 | *Marssonina coronaria* NL1 | KY672995^a^ | OWO98203^a^ | OWP07023^a^ | OWO98660^a^ | OWO98689^a^ |
| 2 | *Marssonina coronaria* YL1 | MT669373 | MT674917 | MT674914 | MT674916 | MT674915 |
| 3 | *Marssonina rosae* | MVNX00000000^b^ | PBP25469^a^ | PBP22390^a^ | PBP23796^a^ | PBP23812^a^ |
| 4 | *Cadophora* sp. DSE1049 | PCYN00000000^b^ | PVH89212^a^ | PVH84997^a^ | PVH80962^a^ | PVH80910^a^ |
| 5 | *Rhynchosporium commune* UK7 | KU844332^a^ | CZS88145^a^ | CZS98640^a^ | CZT11854^a^ | FJUW01000000^b^ |
| 6 | *Chlorociboria aeruginascens* DSM 107184 | MK480517^a^ | TAQ90851^a^ | TAQ85962^a^ | TAQ86149^a^ | TAQ86401^a^ |
| 7 | *Glarea lozoyensis* ATCC 20868 | ALVE01000000^b^ | EPE34718^a^ | EHL01016^a^ | EHK98894^a^ | EPE27098^a^ |
| 8 | *Marssonina brunnea* f. sp. *'multigermtubi'* | JN172909^a^ | EKD17104^a^ | EKD14630^a^ | EKD16333^a^ | EKD16203^a^ |
| 9 | *Phialocephala scopiformis* DAOMC 229536 | LKNI00000000^b^ | KUJ18039^a^ | KUJ23623^a^ | KUJ09066^a^ | KUJ09010^a^ |
| 10 | *Botrytis cinerea* B05.10 | GCF_000143535^b^ | ATZ56955^a^ | ATZ53802^a^ | ATZ51977^a^ | ATZ57660^a^ |
| 11 | *Monilinia fructicola* | VICG00000000^b^ | KAA8564910^a^ | VICG00000000^b^ | KAA8571763^a^ | VICG00000000^b^ |
| 12 | *Ascocoryne sarcoides* | contig_00117:4175-4773^c^ | 6561_t^c^ | 9222_t^c^ | 9149_t^c^ | 9104_t^c^ |
| 13 | *Meliniomyces bicolor* | scaffold_186:1695-2165^c^ | e_gw1.15.578.1^c^ | Locus32v1rpkm1425.58 ^c^ | Locus936v1rpkm149.88 ^c^ | fgenesh1_pm.118_#_64 ^c^ |
| 14 | *Sclerotinia sclerotiorum* | Supercontig_2.35:2419-34401^c^ | SS1G_00885^c^ | SS1G_05520^c^ | SS1G_02087^c^ | SS1G_07798^c^ |
| 15 | *Hymenoscyphus fraxineus* | FXXO00000000^b^ | FXXO00000000^b^ | FXXO00000000^b^ | FXXO00000000^b^ | FXXO00000000^b^ |
| 16 | *Blumeria graminis* f. sp. *hordei* DH14 | CAUH00000000^b^ | CCU77176^a^ | CCU75376^a^ | CCU80781^a^ | CCU80715^a^ |

a, Accession number in Genbank

b, Accession number of genomic assembly. The ITS and gene sequences were obtained by homologous alignment with genomic assembly.

c, Scaffold or locus name in JGI MycoCosm.
