# Supplementary material for: Draft genome sequence of Marssonina coronaria, causal agent of apple blotch, and comparisons with the Marssonina brunnea and Marssonina rosae genomes
Source: PLoS One. 2021 Feb 5;16(2):e0246666. doi: 10.1371/journal.pone.0246666 (PMC7864672; doi:10.1371/journal.pone.0246666)
Supplement: S3 Table — (DOCX) [file pone.0246666.s004.docx]

**S3 Table.** The summary of CAZymes of thirty-six phytopathogenic fungi

| Species | Resource | Lifestyle | PL | | GT^b^ | GH^b^ | CE^b^ | CBM^b^ | AA^b^ | The sum of CAZymes |
| --- | --- | --- | --- | --- | --- | --- | --- | --- | --- | --- |
|  |  |  | PL1-PL36 | Sum |  |  |  |  |  |  |
| *Blumeria graminis* | CAUH00000000^a^ | Biotrophic | NA | 0 | 78 | 90 | 21 | 10 | 14 | 213 |
| *Cladonia grayi* | JGI | Biotrophic | NA | 0 | 111 | 127 | 26 | 12 | 58 | 334 |
| *Tuber melanosporum* | CABJ00000000^a^ | Biotrophic | PL1: 2; PL4: 1 | 3 | 73 | 99 | 22 | 7 | 38 | 242 |
| *Puccinia triticina* | JGI | Biotrophic | PL1: 4; PL35: 1 | 5 | 113 | 163 | 49 | 9 | 38 | 377 |
| *Ustilago maydis* | JGI | Biotrophic | PL1: 1; PL35: 1 | 2 | 86 | 121 | 42 | 11 | 30 | 292 |
| *Melampsora laricis-populina* | JGI | Biotrophic | PL1: 4; PL14: 2; PL35: 1 | 7 | 91 | 183 | 63 | 11 | 50 | 405 |
| *Puccinia graminis* | JGI | Biotrophic | PL1: 2; PL35: 1 | 3 | 98 | 163 | 44 | 10 | 29 | 347 |
| *Laccaria bicolor* | JGI | Biotrophic | PL8: 1; PL14: 6; PL35: 1 | 8 | 100 | 184 | 39 | 20 | 66 | 417 |
| *Cladosporium fulvum* | JGI | Biotrophic | PL1: 3; PL3: 3; PL4: 2; PL7: 1; PL26: 1 | 10 | 133 | 306 | 81 | 23 | 91 | 644 |
| *Phialocephala scopiformis* | LKNI00000000^a^ | Biotrophic | PL1: 1; PL3: 1; PL4: 1; PL35: 2 | 5 | 154 | 425 | 144 | 35 | 192 | 955 |
| *Mycosphaerella graminicola* | JGI | Hemibiotrophic | PL1: 2; PL3: 1 | 3 | 128 | 224 | 58 | 29 | 63 | 505 |
| *Magnaporthe oryzae* | JGI | Hemibiotrophic | PL1: 2; PL3: 1; PL4: 1; PL20: 1; PL26: 1 | 6 | 131 | 293 | 86 | 40 | 29 | 585 |
| *Dothistroma septosporum* | JGI | Hemibiotrophic | PL1: 1; PL4: 2; PL36: 1 | 4 | 136 | 221 | 56 | 20 | 69 | 506 |
| *Moniliophthora perniciosa* | ABRE00000000^a^ | Hemibiotrophic | PL1: 10; PL3: 3; PL4: 1; PL35: 2 | 16 | 47 | 168 | 31 | 7 | 93 | 362 |
| *Colletotrichum graminicola* | JGI | Hemibiotrophic | PL1: 7; PL3: 4; PL4: 3; PL9: 1; PL26: 1; PL27: 1 | 17 | 116 | 327 | 93 | 30 | 145 | 728 |
| *Cochliobolus sativus* | JGI | Hemibiotrophic | PL1: 6; PL3: 5; PL4: 4; PL26: 1 | 16 | 123 | 291 | 82 | 29 | 134 | 675 |
| *Fusarium graminearum* | JGI | Hemibiotrophic | PL1: 9; PL3: 7; PL4: 3; PL9: 1; PL20: 1; PL26: 1 | 22 | 135 | 293 | 89 | 46 | 116 | 701 |
| *Marssonina coronaria* | MZNU00000000^a^ | Hemibiotrophic | PL1: 9; PL3: 6; PL4: 2; PL9: 1; PL20: 1; PL26: 2 | 21 | 116 | 178 | 56 | 24 | 75 | 470 |
| *Marssonina brunea* | AFXC00000000^a^ | Hemibiotrophic | PL1: 9; PL3: 6; PL4: 2; PL9: 2; PL20: 1; PL26: 1 | 21 | 112 | 211 | 60 | 22 | 81 | 507 |
| *Marssonina rosae* | MVNX00000000^a^ | Hemibiotrophic | PL1: 16; PL3: 8; PL4: 7; PL26: 2 | 33 | 194 | 303 | 84 | 33 | 115 | 762 |
| *Rhynchosporium commune* | FJUW00000000^a^ | Hemibiotrophic | PL1: 2; PL3: 3; PL4: 3; PL20: 1; PL26: 1 | 10 | 117 | 286 | 73 | 28 | 96 | 610 |
| *Sclerotinia sclerotiorum* | JGI | Necrotrophic | PL1: 4; PL7: 1 | 5 | 114 | 256 | 63 | 29 | 81 | 548 |
| *Gaeumannomyces graminis* | JGI | Necrotrophic | PL1: 3; PL3: 1; PL4: 1; PL20: 1; PL26: 1 | 7 | 120 | 288 | 80 | 26 | 123 | 644 |
| *Magnaporthe poae* | JGI | Necrotrophic | PL1: 3; PL3: 1; PL4: 1; PL20: 1; PL26: 1 | 7 | 102 | 263 | 59 | 21 | 96 | 548 |
| *Fomitiporia mediterranea* | JGI | Necrotrophic | PL1: 1; PL4: 4; PL35: 1 | 6 | 83 | 212 | 56 | 13 | 97 | 467 |
| *Heterobasidion annosum* | JGI | Necrotrophic | PL1: 2; PL4: 1; PL8: 2; PL14: 4; PL26: 1; PL35: 1 | 11 | 81 | 187 | 53 | 12 | 109 | 453 |
| *Stagonospora nodorum* | JGI | Necrotrophic | PL1: 4; PL3: 2; PL4: 4 | 10 | 108 | 287 | 86 | 19 | 141 | 651 |
| *Pyrenophora tritici-repentis* | JGI | Necrotrophic | PL1: 3; PL3: 3; PL4: 4 | 10 | 116 | 262 | 71 | 24 | 130 | 613 |
| *Botrytis cinerea* B05.10 | AAID00000000^a^ | Necrotrophic | PL1: 7; PL3: 2; PL7: 1 | 10 | 150 | 324 | 85 | 31 | 117 | 717 |
| *Dichomitus squalens* | JGI | Necrotrophic | PL4: 1; PL8: 3; PL14: 7; PL35: 3 | 14 | 82 | 233 | 64 | 18 | 109 | 520 |
| *Fusarium oxysporum* | JGI | Necrotrophic | PL1: 10; PL3: 7; PL4: 4; PL9: 2; PL11: 1; PL26: 1 | 25 | 239 | 528 | 161 | 118 | 202 | 1273 |
| *Fusarium verticillioides* | JGI | Necrotrophic | PL1: 11; PL3: 7; PL4: 3; PL9: 2; PL26: 1; PL29: 1 | 25 | 184 | 420 | 141 | 53 | 147 | 970 |
| *Nectria haematococca* | ACJF00000000^a^ | Necrotrophic | PL1: 14; PL3: 11; PL4: 6; PL7: 1; PL9: 1; PL11: 1; PL20: 1; PL26: 2 | 37 | 146 | 373 | 131 | 63 | 147 | 897 |
| *Verticillium albo-atrum* | JGI | Necrotrophic | PL1: 16; PL3: 11; PL4: 4; PL9: 2; PL11: 1; PL26: 1 | 35 | 112 | 285 | 73 | 25 | 104 | 634 |
| *Verticillium dahliae* | JGI | Necrotrophic | PL1: 16; PL3: 11; PL4: 4; PL9: 2; PL11: 1; PL26: 1 | 35 | 118 | 292 | 78 | 31 | 107 | 661 |
| *Monilinia fructicola* | VICG00000000^a^ | Necrotrophic | PL1: 6; PL3: 1 | 7 | 127 | 263 | 72 | 21 | 98 | 588 |

a, Accession number in Genbank

b, GH, glycoside hydrolase; PL, polysaccharide lyase; CE, carbohydrate esterase; GT, glycosyltransferase; AA, auxiliary activities; CBM, non-catalytic carbohydrate-binding modules.
