# Supplementary material for: Draft genome sequence of Marssonina coronaria, causal agent of apple blotch, and comparisons with the Marssonina brunnea and Marssonina rosae genomes
Source: PLoS One. 2021 Feb 5;16(2):e0246666. doi: 10.1371/journal.pone.0246666 (PMC7864672; doi:10.1371/journal.pone.0246666)
Supplement: S4 Table — (DOCX) [file pone.0246666.s005.docx]

**S4 Table.** Species-specific CAZymes of *M. coronaria*, *M. brunnea* and *M. rosae*.

| CAZyme family^a^ | Gene locus/ name | Potential substrate^b^ | Enzyme activity^b^ | Taxonomy of the top10 best hits^c^ |
| --- | --- | --- | --- | --- |
| *M.coronaria*^b^ | | | | |
| GH3 | B2J93_4547 | Cellulose  Hemicellulose  Pectin | β-glucosidase  β-xylosidase  exo-β-1,4-glucanase | L 9, D 1 |
| GH16 | B2J93_7882 | Hemicellulose | Xyloglucanase | L 9, D 1 |
| GH16 | B2J93_9182 | Hemicellulose | Xyloglucanase | L 2, D 7, S 1 |
| GH18 | B2J93_4402 | N-linked oligosaccharides | endo-β-N-acetylglucosaminidase | L 4, D 3, X 1, E 1, C 1 |
| GH89 | B2J93_2177 | N-linked oligosaccharides | α-N-acetylglucosaminidase | L 9, D 1 |
| GH92 | B2J93_5902 | Oligosaccharides | α-1,2-mannosidase | L 8, D 1, E 1 |
| PL1 | B2J93_4840 | pectin | pectate lyase  exo-pectate lyase | L 5, D 2, E 2, S 1 |
| PL3 | B2J93_5418 | Pectin | Pectate lyase | L 1, D 7, S 2 |
| PL26 | B2J93_9264 | pectin | rhamnogalacturonan exolyase | L 3, S 3, E 3, D 1 |
| CE2 | B2J93_2098 | hemicellulose | acetyl xylan esterase | L 3, D 7 |
| CE4 | B2J93_6888 | Hemicellulose  N-linked oligosaccharides | acetyl xylan esterase  peptidoglycan GlcNAc deacetylase | L 7, D 3 |
| AA1 | B2J93_5665 | lignin | Multicopper oxidases | L 5, S 4, D 1 |
| AA2 | B2J93_2861 | Lignin | Lignin peroxidase | L 2, D 7, S 1 |
| AA3 | B2J93_7165 | Cellulose  lignin | glucose 1-oxidase  aryl alcohol oxidase | L 6, E 3, D 1 |
| AA3 | B2J93_1229 | Cellulose  lignin | glucose 1-oxidase  aryl alcohol oxidase | L 4, D 6 |
| AA3 | B2J93_3628 | Cellulose  lignin | glucose 1-oxidase  aryl alcohol oxidase | D 10 |
| AA7 | B2J93_6765 | Cellobiose  chitin/glycoproteins | glucooligosaccharide oxidase  chitooligosaccharide oxidase | L 2, D 3, S 5 |
| AA7 | B2J93_6390 | See above | See above | L 3, S 6, D 1 |
| AA12 | B2J93_4172 | cellulose | pyrroloquinoline quinone-dependent oxidoreductase | L 9, D 1 |
| GT1 | B2J93_5387 | NA | NA | L 10 |
| GT2 | B2J93_7586 | NA | NA | L 6, D 4 |
| GT31 | B2J93_7585 | NA | NA | L 6, D 4 |
| GT69 | B2J93_6811 | NA | NA | L 4, D 6 |
| GT71 | B2J93_8885 | NA | NA | L 10 |
| *M．brunnea*^b^ | | | | |
| GH1 | MBM_02089 | cellulose  hemicellulose  pectin | β-glucosidase  exo-β-1,4-glucanase  β-galactosidase | L 9, E 1 |
| GH3 | MBM_00209 | Cellulose  Hemicellulose  Pectin | β-glucosidase  β-xylosidase  exo-β-1,4-glucanase | L 4, D 5, E 1 |
| GH3 | MBM_09941 | Cellulose  Hemicellulose  Pectin | β-glucosidase  β-xylosidase  exo-β-1,4-glucanase | L 10 |
| GH3 | MBM_05263 | Cellulose  Hemicellulose  Pectin | β-glucosidase  β-xylosidase  exo-β-1,4-glucanase | L 10 |
| GH3 | MBM_06277 | Cellulose  Hemicellulose  Pectin | β-glucosidase  β-xylosidase  exo-β-1,4-glucanase | L 3, A 1, S 3, E 2, D 1 |
| GH3 | MBM_03435 | Cellulose  Hemicellulose  Pectin | β-glucosidase  β-xylosidase  exo-β-1,4-glucanase | L 10 |
| GH5 | MBM_02055 | Cellulose  Hemicellulose  β-1,3-glucans | endo-β-1,4-glucanase  endo-β-1,4-xylanase  glucan β-1,3-glucosidase | L 7, S 3 |
| GH6 | MBM_04333 | cellulose | endo-β-1,4-glucanase  cellobiohydrolase | L 8, D 2 |
| GH10 | MBM_03633 | hemicellulose | endo-β-1,4-β-xylanase | L 4, E 3, S 3 |
| GH15 | MBM_08967 | Polysaccharides | Glucoamylase | L 10 |
| GH16 | MBM_08515 | Hemicellulose | Xyloglucanase | L 10 |
| GH16 | MBM_00319 | Hemicellulose | Xyloglucanase | L 10 |
| GH28 | MBM_02037 | Pectin | Polygalacturonase | L 1, D 8, E 1 |
| GH28 | MBM_03901 | Pectin | Polygalacturonase | L 10 |
| GH30 | MBM_04774 | cellulose  hemicellulose  pectin | β-glucosidase  endo-β-1,4-xylanase  endo-β-1,6-galactanase | L 6, D 3, S 1 |
| GH30 | MBM_08263 | cellulose  hemicellulose  pectin | β-glucosidase  endo-β-1,4-xylanase  endo-β-1,6-galactanase | L 4, S 5, D 1 |
| GH31 | MBM_03122 | Hemicellulose | α-xylosidase | P 1, D 6, E 2, S 1 |
| GH35 | MBM_08774 | Hemicellulose  Pectin | β-galactosidase  exo-β-1,4-galactanase | L 10 |
| GH35 | MBM_05705 | Hemicellulose  Pectin | β-galactosidase  exo-β-1,4-galactanase | L 9, D 1 |
| GH39 | MBM_08295 | hemicellulose | β-xylosidase | L 9, E 1 |
| GH43 | MBM_04126 | Hemicellulose  Pectin | β-xylosidase  α-L-arabinofuranosidase | L 1, S 9 |
| GH43 | MBM_01191 | Hemicellulose  Pectin | β-xylosidase  α-L-arabinofuranosidase | L 7, E 1, S 1, D 1 |
| GH43 | MBM_05474 | Hemicellulose  Pectin | β-xylosidase  α-L-arabinofuranosidase | L 4, D 2, E 2, S 2 |
| GH43 | MBM_00969 | Hemicellulose  Pectin | β-xylosidase  α-L-arabinofuranosidase | L 8, E 1, D 1 |
| GH43 | MBM_01508 | Hemicellulose  Pectin | β-xylosidase  α-L-arabinofuranosidase | L 6, E 4 |
| GH55 | MBM_03699 | Polysaccharides | endo-1,3-β-glucosidase | L 5, E 5 |
| GH67 | MBM_06928 | Hemicelluloses | α-glucuronidase | L 5, D 4, S 1 |
| GH74 | MBM_05691 | Cellulose  Hemicellulose | endo-β-1,4-glucanase  Xyloglucanase | L 5, T 1, E 2, S 1, Bacteria 1 |
| GH81 | MBM_01851 | Polysaccharides | endo-1,3-β-glucosidase | L 10 |
| GH105 | MBM_04106 | Pectin | rhamnogalacturonyl hydrolase | L 1, S 4, D 5 |
| GH135 | MBM_09138 | exopolysaccharide galactosaminogalactan | α-1,4-galactosaminogalactan hydrolase | L 9, S 1 |
| CE1 | MBM_05316 | Hemicellulose | Acetyl xylan esterase  Feruloyl esterase | L 10 |
| CE3 | MBM_01585 | hemicellulose | acetyl xylan esterase | L 5, S 2, D 1, bacteria 2 |
| CE5 | MBM_09546 | Hemicellulose  Cutin | Acetyl xylan esterase  Cutinase | L 9, E 1 |
| CE5 | MBM_02401 | Hemicellulose  Cutin | Acetyl xylan esterase  Cutinase | L 8, D 1, B 1 |
| CE5 | MBM_03163 | Hemicellulose  Cutin | Acetyl xylan esterase  Cutinase | L 4, S 3, O 2, P 1 |
| CE8 | MBM_01176 | pectin | pectin methylesterase | L 10 |
| CE8 | MBM_00548 | pectin | pectin methylesterase | L 8, D 2 |
| CE10 | MBM_03986 | NA | NA | L 9, E 1 |
| CE10 | MBM_02486 | NA | NA | L 2, D 4, E 4 |
| CE10 | MBM_06621 | NA | NA | L 10 |
| CE10 | MBM_08671 | NA | NA | L 2, E 4, S 3, D 1 |
| CE10 | MBM_03188 | NA | NA | L 10 |
| CE12 | MBM_05265 | Pectin | Pectin acetylesterase | L 2,D 8 |
| AA1 | MBM_02253 | lignin | multicopper oxidases | L 9, S 1 |
| AA3 | MBM_09150 | Cellulose  lignin | glucose 1-oxidase  aryl alcohol oxidase | L 7, D 3 |
| AA3 | MBM_04141 | Cellulose  lignin | glucose 1-oxidase  aryl alcohol oxidase | L 7, D 2, E 1 |
| AA3 | MBM_07353 | Cellulose  lignin | glucose 1-oxidase  aryl alcohol oxidase | L 4, E 5, D 1 |
| AA3 | MBM_08750 | Cellulose  lignin | glucose 1-oxidase  aryl alcohol oxidase | D 10 |
| AA4 | MBM_06330 | Lignin | vanillyl-alcohol oxidase | L 5, E 4, D 1 |
| AA7 | MBM_02730 | Cellobiose  chitin/glycoproteins | glucooligosaccharide oxidase  chitooligosaccharide oxidase | L 1, S 8, E 1 |
| AA7 | MBM_04037 | Cellobiose  chitin/glycoproteins | glucooligosaccharide oxidase  chitooligosaccharide oxidase | S 5, Pis 1, B 1, D 1, E 1 |
| AA7 | MBM_07678 | Cellobiose  chitin/glycoproteins | glucooligosaccharide oxidase  chitooligosaccharide oxidase | L 1, S 3, D 3, E 2, Pis 1 |
| AA7 | MBM_04264 | Cellobiose  chitin/glycoproteins | glucooligosaccharide oxidase  chitooligosaccharide oxidase | L 1, S 8, D 1 |
| AA7 | MBM_03338 | Cellobiose  chitin/glycoproteins | glucooligosaccharide oxidase  chitooligosaccharide oxidase | L 2, E 5, D 2, S 1 |
| AA7 | MBM_04587 | Cellobiose  chitin/glycoproteins | glucooligosaccharide oxidase  chitooligosaccharide oxidase | L 6, E 4 |
| AA7 | MBM_00406 | Cellobiose  chitin/glycoproteins | glucooligosaccharide oxidase  chitooligosaccharide oxidase | L 7, E 2, C 1 |
| AA9 | MBM_00975 | cellulose | copper-dependent monooxygenase | L 10 |
| GT17 | MBM_00251 | NA | NA | L 10 |
| *D. rosea*^b^ | | | | |
| GH53 | PBP24709  PBP28278 | pectin | endo-β-1,4-galactanase | L 10 |
| PL4 | PBP19949  PBP28439 | pectin | rhamnogalacturonan lyase | L 7, D 2, E 1 |
| CE16 | PBP27316  PBP26860 | Polysaccharides | Acetylesterase | L 10 |
| AA2 | PBP21410 | See above | See above | L 10 |
| AA3 | PBP21841 | Cellulose  lignin | glucose 1-oxidase  aryl alcohol oxidase | L 1, S 2, D 5, E 2 |
| CBM48 | PBP22087 | NA | NA | L 10 |
| CBM48 | PBP22865 | NA | NA | L 1/10, D 9/10 |

a, The CAZyme family was annotated by dbcan2 server

b, The potential substrate and enzyme activity were annotated based on two references[1, 2]

c, E, Eurotiomycetes, S, Sordariomycetes, L, Leotiomycetes, D, Dothideomycetes, X, Xylonomycetes, Pis, Pezizomycotina incertae sedis, C, Lecanoromycetes, B, Basidiomycota; P, Pezizomycetes, O, Orbiliomycetes, A, Saccharomycetes, T, Tremellomycetes

Gray filling indicate atypically distributed genes.

1. Blackman LM, Cullerne DP, Hardham AR: **Bioinformatic characterisation of genes encoding cell wall degrading enzymes in the Phytophthora parasitica genome**. *Bmc Genomics* 2014, **15**.

2. Chang HX, Yendrek CR, Caetano-Anolles G, Hartman GL: **Genomic characterization of plant cell wall degrading enzymes and in silico analysis of xylanses and polygalacturonases of Fusarium virguliforme**. *Bmc Microbiol* 2016, **16**.
