# Supplementary material for: Draft genome sequence of Marssonina coronaria, causal agent of apple blotch, and comparisons with the Marssonina brunnea and Marssonina rosae genomes
Source: PLoS One. 2021 Feb 5;16(2):e0246666. doi: 10.1371/journal.pone.0246666 (PMC7864672; doi:10.1371/journal.pone.0246666)
Supplement: S5 Table — (DOCX) [file pone.0246666.s006.docx]

**S5 Table.** The summary of core synthases of secondary metabolism in *Marssonina* spp.

| Enzyme | *M. coronaria* | *M. brunnea* | *M. rosae*^a^ | Potential product | Taxonomy of the top10 best hits^b^ |
| --- | --- | --- | --- | --- | --- |
| PKS1 | B2J93_8989 | MBM_08316 | PBP25820  PBP24039 |  | L 8, D 2 |
| PKS2 | B2J93_6176 | MBM_07653 | PBP19417  PBP17984 | Melanin | L 10 |
| PKS3 | B2J93_9165 | MBM_06369 | PBP18793  PBP18842 |  | L 9, D 1 |
| PKS4 | B2J93_7811 | MBM_06724 | PBP26032  PBP26113 |  | L 3, E 5, S 1, D 1 |
| PKS5 | B2J93_7812 | MBM_06725 | PBP25994  PBP26116 |  | L 2, E 6, S 2 |
| PKS6 | B2J93_9073 | MBM_09202 | PBP26349  PBP22184 |  | L 9, D 1 |
| PKS7 | B2J93_3706 | MBM_00260 | PBP24177  PBP16621 | Melanin | L 9, D 1 |
| PKS8 |  | MBM_04019 |  |  | L 5, E 2, S 2, D 1 |
| PKS9 |  |  | PBP25423  PBP21839 |  | L 1, S 4, E 3, D 2 |
| PKS-NRPS1 |  |  | PBP23442 |  | L 1, S 5, E 4 |
| PKS-NRPS2 | B2J93_2131 | MBM_05146 | PBP20217  PBP17268 | Fusarin | L 3, E 3, S 4 |
| PKS-NRPS3 | B2J93_6983 |  |  |  | D 4, S 3, X 1, Pis 1, E 1 |
| NRPS1 | B2J93_7963 | MBM_09147 | PBP26745  PBP27661 |  | L 8, D 1, E 1 |
| NRPS2 | B2J93_1062 |  |  |  | L 5, S 2, E 2, D 1 |
| NRPS3 | B2J93_4402 |  |  |  | L 4, D 3, X 1, C 1, E 1 |
| NRPS4 | B2J93_3214 | MBM_08979 | PBP21671  PBP20322 |  | L 9, D 1 |
| NRPS5 | B2J93_1626 |  |  |  | L 8, D 2 |
| NRPS6 | B2J93_5257 | MBM_04237 | PBP23436  PBP15752 |  | L 8, D 1, C 1 |
| NRPS7 | B2J93_6044 | MBM_00764 | PBP18462  PBP15667 |  | L 9, D 1 |
| NRPS8 |  | MBM_06951 |  |  | L 7, D 2, E 1 |
| Tc1 |  | MBM_07677 |  | PR toxin | L 1, S 4, D 4, E 1 |
| Tc2 | B2J93_5938 | MBM_04689 | PBP21984  PBP21489 |  | L 9, D 1 |
| Tc3 | B2J93_9055 | MBM_09225 | PBP26350  PBP22206 |  | L 9, D 1 |
| Tc4 |  | MBM_04258 |  |  | L 2, S 4, D 2, E 2 |
| Tc5 |  | MBM_08380 |  |  | L 5, S 2, E 2, D 1 |
| Tc6 | B2J93_8743 | MBM_00659 | PBP17123  PBP28571 |  | L 9, D 1 |
| Tc7 | B2J93_6506 |  |  |  | L 3, B 6, D 1 |
| Tc8 | B2J93_7569 |  | PBP27168  PBP28882 |  | L 3, D 2, S 3, E 1, C 1 |

a, two proteins of *M. rosae* with a continuous identical amino acids≥15 were considered as one pair of duplicated proteins.

b, Blastp against NCBI NR database with core synthase of *M. coronaria* or species-specific core synthase of *M. brunnea* / *M. rosae*. The hits from one genus were counted only once. E, Eurotiomycetes; S, Sordariomycetes; L, Leotiomycetes; D, Dothideomycetes; X, Xylonomycetes; Pis, Pezizomycotina incertae sedis; C, Lecanoromycetes; B, Basidiomycota.
