# Supplementary material for: Draft genome sequence of Marssonina coronaria, causal agent of apple blotch, and comparisons with the Marssonina brunnea and Marssonina rosae genomes
Source: PLoS One. 2021 Feb 5;16(2):e0246666. doi: 10.1371/journal.pone.0246666 (PMC7864672; doi:10.1371/journal.pone.0246666)
Supplement: S6 Table — (DOCX) [file pone.0246666.s007.docx]

**S6 Table.** DHN melanin, Fusarin and PR toxin BGCs in *Marssonina* spp.*

| DHN melanin BGCs | | | |
| --- | --- | --- | --- |
| *B. cinerea*^a^ | *M. coronaria*^b^ | *M. brunnea*^c^ | *M. rosae*^d^ |
| BcPKS12 BGC of DHN melanin | | | |
| BcPKS12  BCIN_02g08770 | B2J93_3706  66% | MBM_00260  68% | PBP24177 67%  PBP16621 66% |
| Bcsmr1  BCIN_02g08760 | B2J93_3704  58% | MBM_00257  57% | PBP24181 33%  PBP16624 66% |
| BcPKS13 BGC of DHN melanin | | | |
| BcPKS13  BCIN_03g08050 | B2J93_6176  62% | MBM_07653  62% | PBP19417 62%  PBP17984 62% |
| Bcztf2  BCIN_03g08080 |  | MBM_07650  40% | PBP16066  41% |
| Bcztf1  BCIN_03g08090 |  |  |  |
| Bcbrn2  BCIN_03g08100 | B2J93_6173  67% | MBM_07651  67% | PBP15475 67%  PBP16067 67% |
| Bcscd1  BCIN_03g08110 | B2J93_6175  58% | MBM_07652  60% | PBP22908 49%  PBP17985 60% |
| Fusarin BGC | | | |
| *Fusarium fujikuroi*^a^ | *M. coronaria*^b^ | *M. brunnea*^c^ | *M. rosae*^d^ |
| fus1 FFUJ_10058 | B2J93__2131  43% | MBM_05146  43% | PBP20217 42%  PBP17268 47% |
| fus2 FFUJ_10057 | B2J93_8919  53% | MBM_07187  53% | PBP16655 53%  PBP25218 54% |
| fus8 FFUJ_10051 |  |  |  |
| fus9 FFUJ_10050 |  |  |  |
| PR toxin BGC | | | |
| *Penicillium chrysogenum*^a^ | *M. coronaria*^b^ | *M. brunnea*^c^ | *M. rosae*^d^ |
| prx2 Pc12g06310 |  | MBM_07677  89% |  |
| prx5 Pc12g06260 |  |  |  |
| prx6 Pc12g06270 |  |  |  |
| prx7 Pc12g06280 |  |  |  |
| prx1 Pc12g06300 |  | MBM_07676  90% |  |
| prx3 Pc12g06320 |  | MBM_07678  81% |  |
| prx4 Pc12g06330 |  | MBM_07679  81% |  |
| prx8 Pc12g06340 |  |  |  |
| prx9 Pc12g06350 |  | MBM_07685  90% |  |
| prx10 Pc12g06360 |  |  |  |
| prx11 Pc12g06370 |  | MBM_07683  83% |  |

*Orthologous relationships between members of model BGC and their homologs of *Marssonina* spp. were confirmed by the reciprocal best hit BLAST.

a, Gene name and locus of members in model BGCs.

b, Gene locus of *M. coronaria* and amino acid identity compared with its ortholog of model BGC.

c, Gene locus of *M. brunnea* and amino acid identity compared with its ortholog of model BGC.

d, Gene locus of *M. rosae* and amino acid identity compared with its ortholog of model BGC.
