# Supplementary material for: Draft genome sequence of Marssonina coronaria, causal agent of apple blotch, and comparisons with the Marssonina brunnea and Marssonina rosae genomes
Source: PLoS One. 2021 Feb 5;16(2):e0246666. doi: 10.1371/journal.pone.0246666 (PMC7864672; doi:10.1371/journal.pone.0246666)
Supplement: S7 Table — (DOCX) [file pone.0246666.s008.docx]

**S7 Table.** The summary of homologs of PKS-NRPS3 of *M. coronaria* used in phylogenetic analysis

| Gene name | Species | Strain/Isolate | Taxonomy | Accession number | E-Value/Identity |
| --- | --- | --- | --- | --- | --- |
| McSwnK-like2 | *Marssonina coronaria* | NL1 | Leotiomycetes | OWP04937 |  |
| TbSwnK | Trichophyton benhamiae | CBS 112371 | Eurotiomycetes | XP_003014124 | 0/52% |
| TvioSwnK | Trichophyton violaceum | CMCC(F)T3l | Eurotiomycetes | OAL75151 | 0/52% |
| TverSwnK | Trichophyton verrucosum | HKI 0517 | Eurotiomycetes | XP_003020763 | 0/52% |
| TeSwnK | Trichophyton equinum | CBS 127.97 | Eurotiomycetes | EGE01982 | 0/52% |
| TiSwnK | Trichophyton interdigitale | H6 | Eurotiomycetes | EZF30502 | 0/52% |
| TiSwnK | Trichophyton interdigitale | MR816 | Eurotiomycetes | KDB22226 | 0/52% |
| TrSwnK | Trichophyton rubrum | CBS 118892 | Eurotiomycetes | XP_003238870 | 0/52% |
| TrSwnK | Trichophyton rubrum | CMCC(F)T1i | Eurotiomycetes | OAL63126 | 0/52% |
| TrSwnK | Trichophyton rubrum | CBS 289.86 | Eurotiomycetes | EZF58570 | 0/52% |
| TrSwnK | Trichophyton rubrum | CBS 735.88 | Eurotiomycetes | EZG01521 | 0/52% |
| TrSwnK | Trichophyton rubrum | CBS 100081 | Eurotiomycetes | EZF37323 | 0/51% |
| TsSwnK | Trichophyton soudanense | CBS 452.61 | Eurotiomycetes | EZF69148 | 0/52% |
| TtSwnK | Trichophyton tonsurans | CBS 112818 | Eurotiomycetes | EGD97139 | 0/51% |
| NgSwnK | Nannizzia gypsea | CBS 118893 | Eurotiomycetes | XP_003176907 | 0/51% |
| McanSwnK | Microsporum canis | CBS 113480 | Eurotiomycetes | XP_002850891 | 0/51% |
| AoSwnK | Alternaria oxytropis |  | Dothideomycetes | AQV04230 | 0/51% |
| SlSwnK1 | Slafractonia leguminicola |  | Pezizomycotina incertae sedis | AQV04236 | 0/51% |
| PspSwnK | Pseudogymnoascus sp. | VKM F-4515 | Leotiomycetes | KFY51099 | 0/52% |
| FspSwnK | Fungal sp. | No.14919 |  | GAW19803 | 0/53% |
| CspSwnK | Chaetothyriaceae sp. |  | Eurotiomycetes | AQV04224 | 0/55% |
| MacrSwnK | Metarhizium acridum | CQMa 102 | Sordariomycetes | XP_007815889 | 0/54% |
| ManiSwnK | Metarhizium anisopliae | ARSEF 549 | Sordariomycetes | KID61008 | 0/54% |
| ManiSwnK | Metarhizium anisopliae | BRIP 53293 | Sordariomycetes | KJK74452 | 0/52% |
| ManiSwnK | Metarhizium anisopliae | BRIP 53284 | Sordariomycetes | KJK85338 | 0/51% |
| ManiSwnK | Metarhizium anisopliae | E6 | Sordariomycetes | KFG82172 | 0/54% |
| MmSwnK | Metarhizium majus | ARSEF 297 | Sordariomycetes | XP_014575312 | 0/56% |
| MgSwnK | Metarhizium guizhouense | ARSEF 977 | Sordariomycetes | KID83603 | 0/54% |
| MrSwnK | Metarhizium robertsii | ARSEF 2575 | Sordariomycetes | EXU97982 | 0/53% |
| MrSwnK | Metarhizium robertsii | ARSEF 23 | Sordariomycetes | XP_007824811 | 0/53% |
| MbSwnK | Metarhizium brunneum | ARSEF 3297 | Sordariomycetes | XP_014543166 | 0/54% |
| SlSwnK2 | Slafractonia leguminicola |  | Pezizomycotina incertae sedis | AQV04238 | 0/51% |
| MspSwnK-like1 | Monosporascus sp. | MG133 | Sordariomycetes | RYP40702 | 0/58% |
| MspSwnK-like1 | Monosporascus sp. | CRB-9-2 | Sordariomycetes | RYP63027 | 0/58% |
| MspSwnK-like1 | Monosporascus sp. | GIB2 | Sordariomycetes | RYP05213 | 0/59% |
| MspSwnK-like1 | Monosporascus sp. | CRB-8-3 | Sordariomycetes | RYP55625 | 0/58% |
| PvSwnK-like1 | Pseudomassariella vexata | CBS 129021 | Sordariomycetes | ORY60966 | 0/59% |
| EpSwnk-like1 | Endocarpon pusillum | Z07020 | Eurotiomycetes | XP_007805680 | 0/58% |
| ElSwnk-like1 | Eutypa lata | UCREL1 | Sordariomycetes | EMR62731 | 0/58% |
| CgSwnK-like2 | Cenococcum geophilum | 1.58 | Dothideomycetes | OCL00726 | 0/64% |
| XhSwnK-like2 | Xylona heveae | TC161 | Xylonomycetes | XP_018192493 | 0/63% |
| MspSwnK-like2 | Monosporascus sp. | mg162 | Sordariomycetes | RYP50631 | 0/63% |
| SlSwnK-like2 | Slafractonia leguminicola |  | Pezizomycotina incertae sedis | AQV04237 | 0/62% |
| BoSwnK-like2 | Bipolaris oryzae | ATCC 44560 | Dothideomycetes | XP_007689897 | 0/58% |
| BmSwnK-like2 | Bipolaris maydis | ATCC 48331 | Dothideomycetes | XP_014076613 | 0/58% |
| BsSwnK-like2 | Bipolaris sorokiniana | ND90Pr | Dothideomycetes | XP_007704421 | 0/58% |
| BvSwnK-like2 | Bipolaris victoriae | FI3 | Dothideomycetes | XP_014553644 | 0/58% |
| BzSwnK-like2 | Bipolaris zeicola | 26-R-13 | Dothideomycetes | XP_007711317 | 0/58% |
