# Supplementary material for: Draft genome sequence of Marssonina coronaria, causal agent of apple blotch, and comparisons with the Marssonina brunnea and Marssonina rosae genomes
Source: PLoS One. 2021 Feb 5;16(2):e0246666. doi: 10.1371/journal.pone.0246666 (PMC7864672; doi:10.1371/journal.pone.0246666)
Supplement: S8 Table — (DOCX) [file pone.0246666.s009.docx]

**S8 Table.** The summary of the small secreted proteins of *Marssonina coronaria*

| Gene name | *Marssonina coronaria* | *Marssonina*  *brunnea* | *Marssonina rosae* | Taxonomy of the first hit^a^ | Taxonomy of the top10 best hits ^a^ | Description ^b^ | Cysteine residue |
| --- | --- | --- | --- | --- | --- | --- | --- |
| McSSP1 | B2J93_6771 | MBM_00915# | PBP27545#  PBP26758# | L | L 10 | FKBP-type peptidyl-prolyl cis-trans isomerase | 2 |
| McSSP2 | B2J93_4143 | MBM_04682 | PBP25468  PBP21420 | L | L 10 | PET assembly of cytochrome c oxidase, mitochondrial | 0 |
| McSSP3 | B2J93_7733 | MBM_06153# | PBP19571# | L | L 10 | WSC domain | 9 |
| McSSP4 | B2J93_1682 | MBM_07761# | PBP20890#  PBP25111# | L | L 10 | Egh16-like virulence factor | 6 |
| McSSP5 | B2J93_5181 | MBM_02478# | PBP19448#  PBP15643# | L | L 10 | Cerato-platanin | 4 |
| McSSP6 | B2J93_6784 | MBM_05034# | PBP21731#  PBP22229# | L | L 10 | emp24/gp25L/p24 family/GOLD | 3 |
| McSSP7 | B2J93_7320 | MBM_01562 | PBP24325  PBP29012 | L | L 10 | ADP-ribosylation factor family | 3 |
| McSSP8 | B2J93_5326 | MBM_05118# | PBP21744#  PBP22266# | L | L 10 | CFEM domain | 8 |
| McSSP9 | B2J93_2777 | MBM_07010# | PBP18451#  PBP18548# | L | L 9; D 1 | Plastocyanin-like domain | 3 |
| McSSP10 | B2J93_3295 | MBM_03014 | PBP26068  PBP26093 | L | L 9; D 1 | RecQ mediated genome instability protein | 2 |
| McSSP11 | B2J93_9069 | MBM_09206# | PBP22198# | L | L 9; D 1 |  | 4 |
| McSSP12 | B2J93_7269 | MBM_04851# | PBP25837# | L | L 9; D 1 |  | 15 |
| McSSP13 | B2J93_8055 | MBM_08458 | PBP23382# | L | L 9; D 1 | Ubiquitin 3 binding protein But2 C-terminal domain | 4 |
| McSSP14 | B2J93_8975 | MBM_06150 | PBP24044  PBP25767 | L | L 9; D 1 |  | 4 |
| McSSP15 | B2J93_888 | MBM_06958# | PBP28259#  PBP24651# | L | L 9; D 1 |  | 3 |
| McSSP16 | B2J93_3657 | MBM_04124 | PBP21614  PBP26209 | L | L 9; S 1 |  | 0 |
| McSSP17 | B2J93_5650 | MBM_07937# | PBP19028#  PBP18087# | L | L 9; D 1 | Protein of unknown function (DUF3455) | 4 |
| McSSP18 | B2J93_7259 | MBM_04864 | PBP25853# | L | L 9; D 1 | Cysteine-rich secretory protein family | 5 |
| McSSP19 | B2J93_7651 | MBM_09400# | PBP16508# | L | L 9; D 1 |  | 3 |
| McSSP20 | B2J93_8195 | MBM_02168# | PBP21457#  PBP25519# | L | L 9; D 1 | Killer toxin-resistance protein 1 | 0 |
| McSSP21 | B2J93_229 | MBM_05824# | PBP27322#  PBP26952# | L | L 9; D 1 |  | 10 |
| McSSP22 | B2J93_5015 | MBM_08162# | PBP16773# | L | L 9; D 1 |  | 3 |
| McSSP23 | B2J93_7289 | MBM_04245 | PBP25498# | L | L 9; D 1 |  | 4 |
| McSSP24 | B2J93_2545 | MBM_02262 | PBP28164# | L | L 9; D1 |  | 2 |
| McSSP25 | B2J93_9 | MBM_06307# | PBP22992# | L | L 9; D 1 | CFEM domain | 8 |
| McSSP26 | B2J93_846 | MBM_08331 | PBP28277#  PBP27202# | L | L 9; D 1 |  | 2 |
| McSSP27 | B2J93_7010 |  | PBP24136# | L | L 9; D 1 | ML domain | 3 |
| McSSP28 | B2J93_7832 | MBM_08916 | PBP22320  PBP24814 | L | L 8; S 2 | Trypsin | 6 |
| McSSP29 | B2J93_3459 | MBM_06072# | PBP19799# | L | L 8; D 2 | Lytic polysaccharide mono-oxygenase, cellulose-degrading | 7 |
| McSSP30 | B2J93_8634 | MBM_00547# | PBP28475 | L | L 8; D 1; E 1 | Isochorismatase family | 1 |
| McSSP31 | B2J93_1978 | MBM_01783 | PBP17212# | L | L 8; D 1; E 1 |  | 5 |
| McSSP32 | B2J93_3893 | MBM_02183 | PBP20387 | L | L 8; S 1; E 1 | Glycosyl hydrolase family 61 | 4 |
| McSSP33 | B2J93_8559 | MBM_00405# | PBP17512# | Bacteria | L 8; Bacteria 1; D 1 |  | 0 |
| McSSP34 | B2J93_82 | MBM_03332# | PBP20623# | L | L 8; S 2 | Cytochrome C oxidase assembly factor 2 | 3 |
| McSSP35 | B2J93_3655 | MBM_00109# | PBP26280#  PBP21613# | L | L 7; S 3 | Ser-Thr-rich glycosyl-phosphatidyl-inositol-anchored membrane family | 1 |
| McSSP36 | B2J93_7089 | MBM_01252# | PBP23642# | L | L 7; E 2; S 1 | Necrosis inducing protein (NPP1) | 4 |
| McSSP37 | B2J93_2606 | MBM_03813 | PBP18109# | L | L 7; Bacteria 1; D 2 |  | 8 |
| McSSP38 | B2J93_4624 | MBM_04824# | PBP22845#  PBP24558# | L | L 7; D 1 |  | 6 |
| McSSP39 | B2J93_3968 | MBM_02028 | PBP28139# | L | L 7; D 1; S 1; B 1 |  | 3 |
| McSSP40 | B2J93_5511 | MBM_02513 | PBP28323#  PBP18523# | L | L 7; S 2; D 1 | Ser-Thr-rich glycosyl-phosphatidyl-inositol-anchored membrane family | 0 |
| McSSP41 | B2J93_4420 | MBM_09178# | PBP16151#  PBP21179# | Bacteria | L 7; Bacteria 1 |  | 4 |
| McSSP42 | B2J93_5149 | MBM_01372# | PBP18404# | L | L 7; D 3 |  | 9 |
| McSSP43 | B2J93_1400 | MBM_06793 | PBP20828 | L | L 7; C 1 | MIOREX complex component 7 | 1 |
| McSSP44 | B2J93_198 | MBM_06977 | PBP25891  PBP15852 | L | L 6; S 3; D 1 | Glycosyl hydrolase family 61 | 6 |
| McSSP45 | B2J93_1862 | MBM_05302 | PBP24913 | L | L 6; D 3; S 1 |  | 5 |
| McSSP46 | B2J93_4114 | MBM_03877# | PBP22972#  PBP24234# | L | L 6; D 1 |  | 5 |
| McSSP47 | B2J93_7593 | MBM_06694# | PBP28906# | L | L 6; D 1; S 3 |  | 3 |
| McSSP48 | B2J93_4249 | MBM_05777 | PBP16183# | L | L 6; D 1 |  | 6 |
| McSSP49 | B2J93_6838 | MBM_03679# | PBP18492#  PBP24165# | L | L 6 |  | 1 |
| McSSP50 | B2J93_8565 | MBM_08741# | PBP17505# | L | L 5; D 5 |  | 0 |
| McSSP51 | B2J93_689 | MBM_00324 | PBP25403 | L | L 5; S 4; E 1 |  | 14 |
| McSSP52 | B2J93_3620 | MBM_00141# | PBP26264#  PBP21586# | L | L 5; D 2; S 2 | Pectate lyase | 10 |
| McSSP53 | B2J93_2761 | MBM_01819# | PBP18202#  PBP23342# | L | L 4; S 1; D 5 |  | 8 |
| McSSP54 | B2J93_1949 | MBM_00286# | PBP23319#  PBP26284# | L | L 4; D 4; S 2 | Cutinase | 4 |
| McSSP55 | B2J93_9431 | MBM_06673# | PBP26035# | L | L 4; D 3; S 3 |  | 4 |
| McSSP56 | B2J93_5245 | MBM_09508 | PBP15874  PBP22529 | L | L 3; S 5; A 2 |  | 7 |
| McSSP57 | B2J93_8504 | MBM_08351# | PBP23677#  PBP26346# | L | L 3; S 1; B 1 |  | 1 |
| McSSP58 | B2J93_726 | MBM_03198# | PBP21891#  PBP16837# | L | L 3; D 4; S 3 | Cutinase | 4 |
| McSSP59 | B2J93_4556 | MBM_05995 | PBP21809  PBP25425 | D | L 2; D 3; S 5 | Glycosyl hydrolase family 12 | 0 |
| McSSP60 | B2J93_3460 | MBM_06081# | PBP19810#  PBP15903# | L | L 2; S 4; D 2 | Peroxidase, family 2 | 2 |
| McSSP61 | B2J93_5291 | MBM_09907 | PBP18918# | S | L 2; S 3; D 5 |  | 3 |
| McSSP62 | B2J93_6070 | MBM_06273 | PBP28481#  PBP15802# | L | L 2; D 1 | CFEM domain | 8 |
| McSSP63 | B2J93_8986 | MBM_08313# | PBP25818# | L | L 2; D 4; S 3; E 1 | necrosis-inducing secreted protein 1 (NIS1) | 0 |
| McSSP64 | B2J93_1563 | MBM_05194 | PBP25672#  PBP19957# | L | L 1 |  | 2 |
| McSSP65 | B2J93_3131 | MBM_09012 | PBP15588  PBP23715 | L | L 1 |  | 0 |
| McSSP66 | B2J93_499 | MBM_08651# | PBP26827# | S | L 1; S 7; E 1; D 1 | Complex I intermediate-associated protein 30 (CIA30) | 1 |
| McSSP67 | B2J93_2593 | MBM_01161# | PBP20618 | D | L 1; D 8; E 1 | Protein of unknown function (DUF1524) | 4 |
| McSSP68 | B2J93_724 | MBM_08078# | PBP20730# | L | L 1; D 7; S 2 |  | 2 |
| McSSP69 | B2J93_7020 | MBM_02020# | PBP21162# | L | L 1 |  | 7 |
| McSSP70 | B2J93_6424 | MBM_08471# | PBP18894#  PBP21706# | S | L 1; S 5; D 4 |  | 2 |
| McSSP71 | B2J93_988 | MBM_00728# | PBP16090# | Unique | Unique |  | 6 |
| McSSP72 | B2J93_1751 | MBM_07504# |  | L | L 10 |  | 4 |
| McSSP73 | B2J93_278 | MBM_01394 |  | L | L 7; D 1 |  | 0 |
| McSSP74 | B2J93_5314 | MBM_08007# |  | L | L 6; D 1 |  | 4 |
| McSSP75 | B2J93_7203 | MBM_00186 |  | L | L 5; E 3; S 1 | Cutinase | 10 |
| McSSP76 | B2J93_3871 | MBM_08179# |  | L | L 5 | LysM domain | 4 |
| McSSP77 | B2J93_8755 | MBM_04262 |  | L | L 3; D 7 | Membrane-Associated Proteins in Eicosanoid and Glutathione metabolism | 2 |
| McSSP78 | B2J93_5997 | MBM_01882# |  | L | L 2 | HTH domain | 3 |
| McSSP79 | B2J93_1470 | MBM_04139# |  | L | L 1; D 2; S 1 |  | 6 |
| McSSP80 | B2J93_3261 | MBM_03898 |  | Unique | Unique |  | 5 |
| McSSP81 | B2J93_5046 |  | PBP16686# | L | L 10 | Cutinase | 6 |
| McSSP82 | B2J93_3654 |  | PBP21612#  PBP26211# | L | L 9, D 1 | Ser-Thr-rich glycosyl-phosphatidyl-inositol-anchored membrane family | 0 |
| McSSP83 | B2J93_1651 |  | PBP18281# | L | L 8; D 2 |  | 4 |
| McSSP84 | B2J93_3797 |  | PBP24251# PBP23018# | L | L 4; S 2; D 4 | Pectate lyase | 10 |
| McSSP85 | B2J93_1408 |  | PBP20819#  PBP23090# | L | L 4; D 1 |  | 8 |
| McSSP86 | B2J93_3586 |  | PBP28021# | E | L 3; E 3; S 2 | Necrosis inducing protein (NPP1) | 5 |
| McSSP87 | B2J93_1803 |  | PBP19294#  PBP24975# | L | L 1; Slime mold 1; animal 7; plant 1 | Thioredoxin | 6 |
| McSSP88 | B2J93_2874 |  | PBP28136# | E | E 4; D 5; S 1 | Glyoxalase/Bleomycin resistance protein/Dioxygenase superfamily | 1 |
| McSSP89 | B2J93_5049 |  | PBP15843#  PBP18374# | Unique | Unique |  | 2 |
| McSSP90 | B2J93_4738 |  | PBP17358# | Unique | Unique |  | 8 |
| McSSP91 | B2J93_5210 |  | PBP16002# | Unique | Unique |  | 3 |
| McSSP92 | B2J93_8846 |  | PBP19667# | Unique | Unique |  | 7 |
| McSSP93 | B2J93_8548 |  | PBP15722# PBP15509# | Unique | Unique |  | 8 |
| McSSP94 | B2J93_3653 |  | PBP26210# | Unique | Unique |  | 9 |
| McSSP95 | B2J93_2866 |  | PBP28128# | Unique | Unique |  | 0 |
| McSSP96 | B2J93_5791 |  | PBP23928 | Unique | Unique |  | 6 |
| McSSP97 | B2J93_9600 |  | PBP25383# | Unique | Unique |  | 10 |
| McSSP98 | B2J93_7959 |  | PBP28752# | Unique | Unique |  | 8 |
| McSSP99 | B2J93_4225 |  |  | L | L 10 | Membrane magnesium transporter | 2 |
| McSSP100 | B2J93_8124 |  |  | L | L 7; E 2; Bacteria 1 | beta-acetyl hexosaminidase like | 0 |
| McSSP101 | B2J93_7909 |  |  | Bacteria | L 4; D 4; E 1; Bacteria 1 |  | 2 |
| McSSP102 | B2J93_5785 |  |  | L | L 1 |  | 0 |
| McSSP103 | B2J93_6058 |  |  | Unique | Unique |  | 0 |
| McSSP104 | B2J93_9379 |  |  | Unique | Unique | Glycosyl hydrolase catalytic core | 1 |
| McSSP105 | B2J93_3976* |  |  | L | L 9; D 1 |  | 3 |
| McSSP106 | B2J93_602* |  |  | L | L 9; D 1 |  | 2 |
| McSSP107 | B2J93_9089* |  |  | L | L 9; D 1 |  | 1 |
| McSSP108 | B2J93_4386* |  |  | L | L 7; S 3 | Protein of unknown function (DUF3494) | 6 |
| McSSP109 | B2J93_9481* |  |  | L | L 7 |  | 9 |
| McSSP110 | B2J93_86* |  |  | L | L 5; E 3; D 2 |  | 3 |
| McSSP111 | B2J93_3551* |  |  | L | L 5; D 1 |  | 5 |
| McSSP112 | B2J93_2393* |  |  | L | L 4 |  | 0 |
| McSSP113 | B2J93_1239* |  |  | L | L 4 |  | 3 |
| McSSP114 | B2J93_302* |  |  | L | L 3; D 6; O 1 |  | 16 |
| McSSP115 | B2J93_8814* |  |  | L | L 3; S 2; D 5 | Alkaline phosphatase | 1 |
| McSSP116 | B2J93_7233* |  |  | L | L 3; D 3 |  | 6 |
| McSSP117 | B2J93_7530* |  |  | L | L 3; D 1 |  | 8 |
| McSSP118 | B2J93_8305* |  |  | L | L 2; E 1 |  | 6 |
| McSSP119 | B2J93_3372* |  |  | S | L 2; S 7; D 1 |  | 6 |
| McSSP120 | B2J93_5474* |  |  | L | L 2; D 5; E 3 |  | 3 |
| McSSP121 | B2J93_7766* |  |  | L | L 2 |  | 8 |
| McSSP122 | B2J93_224* |  |  | L | L 2 |  | 4 |
| McSSP123 | B2J93_5837* |  |  | L | L 1; D 1 |  | 8 |
| McSSP124 | B2J93_9365* |  |  | L | L 1 |  | 0 |
| McSSP125 | B2J93_6828* |  |  | L | L 1 |  | 2 |
| McSSP126 | B2J93_9483* |  |  | L | L 1 |  | 1 |
| McSSP127 | B2J93_6502* |  |  | L | L 1 |  | 0 |
| McSSP128 | B2J93_5714* |  |  | L | L 1 |  | 8 |
| McSSP129 | B2J93_63* |  |  | S | S 4; O 2; E 2; D 2 |  | 4 |
| McSSP130 | B2J93_3462* |  |  | O | O 1; S 4; D 1 |  | 6 |
| McSSP131 | B2J93_7285* |  |  | S | S 2; D 2 |  | 4 |
| McSSP132 | B2J93_6223* |  |  | D | D 1 |  | 0 |
| McSSP133 | B2J93_5148* |  |  | Unique | Unique |  | 5 |
| McSSP134 | B2J93_6749* |  |  | Unique | Unique |  | 0 |
| McSSP135 | B2J93_2423* |  |  | Unique | Unique |  | 1 |
| McSSP136 | B2J93_9317* |  |  | Unique | Unique |  | 0 |
| McSSP137 | B2J93_134* |  |  | Unique | Unique |  | 0 |
| McSSP138 | B2J93_9183* |  |  | Unique | Unique |  | 0 |
| McSSP139 | B2J93_8807* |  |  | Unique | Unique |  | 1 |
| McSSP140 | B2J93_7126* |  |  | Unique | Unique |  | 0 |
| McSSP141 | B2J93_9312* |  |  | Unique | Unique |  | 2 |
| McSSP142 | B2J93_5766* |  |  | Unique | Unique |  | 4 |
| McSSP143 | B2J93_9558* |  |  | Unique | Unique |  | 6 |
| McSSP144 | B2J93_7574* |  |  | Unique | Unique |  | 2 |
| McSSP145 | B2J93_8166* |  |  | Unique | Unique |  | 9 |
| McSSP146 | B2J93_858* |  |  | Unique | Unique |  | 5 |
| McSSP147 | B2J93_3417* |  |  | Unique | Unique |  | 4 |
| McSSP148 | B2J93_5072* |  |  | Unique | Unique |  | 1 |
| McSSP149 | B2J93_9240* |  |  | Unique | Unique |  | 6 |
| McSSP150 | B2J93_2409* |  |  | Unique | Unique |  | 0 |
| McSSP151 | B2J93_4166* |  |  | Unique | Unique |  | 1 |
| McSSP152 | B2J93_149* |  |  | Unique | Unique |  | 4 |
| McSSP153 | B2J93_2856* |  |  | Unique | Unique |  | 3 |
| McSSP154 | B2J93_8799* |  |  | Unique | Unique |  | 0 |
| McSSP155 | B2J93_2607* |  |  | Unique | Unique |  | 7 |
| McSSP156 | B2J93_8427* |  |  | Unique | Unique |  | 5 |
| McSSP157 | B2J93_3060* |  |  | Unique | Unique |  | 7 |
| McSSP158 | B2J93_6204* |  |  | Unique | Unique |  | 9 |
| McSSP159 | B2J93_5503* |  |  | Unique | Unique |  | 5 |
| McSSP160 | B2J93_3259* |  |  | Unique | Unique |  | 0 |
| McSSP161 | B2J93_9128* |  |  | Unique | Unique |  | 2 |
| McSSP162 | B2J93_3101* |  |  | Unique | Unique |  | 0 |
| McSSP163 | B2J93_5068* |  |  | Unique | Unique |  | 4 |
| McSSP164 | B2J93_6057* |  |  | Unique | Unique |  | 1 |
| McSSP165 | B2J93_5011* |  |  | Unique | Unique |  | 2 |
| McSSP166 | B2J93_6115* |  |  | Unique | Unique |  | 3 |
| McSSP167 | B2J93_1394* |  |  | Unique | Unique |  | 3 |
| McSSP168 | B2J93_796* |  |  | Unique | Unique |  | 1 |
| McSSP169 | B2J93_4212* |  |  | Unique | Unique |  | 6 |
| McSSP170 | B2J93_5852* |  |  | Unique | Unique |  | 3 |
| McSSP171 | B2J93_1269* |  |  | Unique | Unique |  | 4 |
| McSSP172 | B2J93_6500* |  |  | Unique | Unique |  | 0 |
| McSSP173 | B2J93_9458* |  |  | Unique | Unique |  | 5 |
| McSSP174 | B2J93_9486* |  |  | Unique | Unique |  | 2 |
| McSSP175 | B2J93_5623* |  |  | Unique | Unique |  | 1 |
| McSSP176 | B2J93_7141* |  |  | Unique | Unique |  | 2 |
| McSSP177 | B2J93_5673* |  |  | Unique | Unique |  | 0 |
| McSSP178 | B2J93_4131* |  |  | Unique | Unique |  | 1 |
| McSSP179 | B2J93_9620* |  |  | Unique | Unique |  | 2 |
| McSSP180 | B2J93_4238* |  |  | Unique | Unique |  | 3 |
| McSSP181 | B2J93_375* |  |  | Unique | Unique |  | 11 |
| McSSP182 | B2J93_4660* |  |  | Unique | Unique |  | 5 |
| McSSP183 | B2J93_6772* |  |  | Unique | Unique |  | 5 |
| McSSP184 | B2J93_9409* |  |  | Unique | Unique | Protein of unknown function (DUF2895) | 0 |
| McSSP185 | B2J93_1739* |  |  | Unique | Unique |  | 6 |
| McSSP186 | B2J93_123* |  |  | Unique | Unique |  | 6 |
| McSSP187 | B2J93_5998* |  |  | Unique | Unique | Variant SH3 domain | 4 |

a The hits from one genus were counted only once. E, Eurotiomycetes; S, Sordariomycetes; L, Leotiomycetes; D, Dothideomycetes; X, Xylonomycetes; P, Pezizomycotina incertae sedis, C, Lecanoromycetes, O, Orbiliomycetes, A, Saccharomycetes; F, Schizosaccharomycetes; G, Basidiobolomycetes; B, Basidiomycota; Fis, Fungi incertae sedis; Slime mold, Amoebozoa Eumycetozoa; Animal, Metazoa; Plant, Viridiplantae magnoliopsida

Yellow grid, orthologs confirmed by the best-reciprocal-hit Blast

b Protein domain was predicted by Pfam (<http://pfam.xfam.org/>) and blastp in NCBI NR database.

*Species-specific SSPs

# SSPs in *M. brunnea* and *M. rosae*.
