# Supplementary material for: Draft genome sequence of Marssonina coronaria, causal agent of apple blotch, and comparisons with the Marssonina brunnea and Marssonina rosae genomes
Source: PLoS One. 2021 Feb 5;16(2):e0246666. doi: 10.1371/journal.pone.0246666 (PMC7864672; doi:10.1371/journal.pone.0246666)
Supplement: S9 Table — (DOCX) [file pone.0246666.s010.docx]

**S9 Table** The summary of the small secreted proteins of *Marssonina brunnea*

| Gene game | *Marssonina brunnea* | *Marssonina coronaria* | *Marssonina rosae* | Taxonomy of the best hit | Taxonomy of the top10 best hitsa | Description | Cysteine residue |
| --- | --- | --- | --- | --- | --- | --- | --- |
| MbSSP1 | MBM_08076 | B2J93_6267 | PBP25090# | L | L 10 |  | 6 |
| MbSSP2 | MBM_09400 | B2J93_7651# | PBP16508# | L | L 10 |  | 2 |
| MbSSP3 | MBM_07761 | B2J93_1682# | PBP20890# PBP25111# | L | L 9; D 1 | Egh16-like virulence factor | 6 |
| MbSSP4 | MBM_00122 | B2J93_1957 | PBP27498 PBP17470 | D | L 9; D 1 | Protein of unknown function (DUF2012) | 2 |
| MbSSP5 | MBM_05824 | B2J93_229# | PBP27322# PBP26952# | L | L 9; D 1 |  | 10 |
| MbSSP6 | MBM_05877 | B2J93_2694 | PBP20673# PBP27039# | D | L 9; D 1 | emp24/gp25L/p24 family/GOLD | 2 |
| MbSSP7 | MBM_01173 | B2J93_2773 | PBP23338 PBP18545 | D | L 9; D 1 |  | 4 |
| MbSSP8 | MBM_09529 | B2J93_3084 | PBP23248# PBP25685# | D | L 9; D 1 |  | 0 |
| MbSSP9 | MBM_01208 | B2J93_3113 | PBP22726# PBP28729# | D | L 9; D 1 |  | 3 |
| MbSSP10 | MBM_06647 | B2J93_3980 | PBP17496 PBP21096 | L | L 9; D 1 |  | 4 |
| MbSSP11 | MBM_08162 | B2J93_5015# | PBP16773# | D | L 9; D 1 | Cutinase | 5 |
| MbSSP12 | MBM_05118 | B2J93_5326# | PBP21744# PBP22266# | D | L 9; D 1; | CFEM domain | 8 |
| MbSSP13 | MBM_07937 | B2J93_5650# | PBP19028# PBP18087# | D | L 9; D 1 | Protein of unknown function (DUF3455) | 4 |
| MbSSP14 | MBM_01844 | B2J93_5981 | PBP23326 PBP18213 | D | L 9; D 1 |  | 0 |
| MbSSP15 | MBM_00915 | B2J93_6771# | PBP27545# PBP26758# | L | L 9; D 1 | FKBP-type peptidyl-prolyl cis-trans isomerase | 2 |
| MbSSP16 | MBM_05034 | B2J93_6784# | PBP21731# PBP22229# | L | L 9; D 1 | emp24/gp25L/p24 family/GOLD | 3 |
| MbSSP17 | MBM_06153 | B2J93_7733# | PBP19571# | D | L 9; D 1 | WSC domain | 9 |
| MbSSP18 | MBM_03438 | B2J93_7915 | PBP18814# | L | L 9; D 1 |  | 0 |
| MbSSP19 | MBM_02168 | B2J93_8195# | PBP21457# PBP25519# | L | L 9; D 1 | Killer toxin-resistance protein 1 | 0 |
| MbSSP20 | MBM_06958 | B2J93_888# | PBP28259# PBP24651# | L | L 9; D 1 |  | 3 |
| MbSSP21 | MBM_05578 | B2J93_9426 | PBP24590 PBP26133 | L | L 9; D 1 |  | 4 |
| MbSSP22 | MBM_06307 | B2J93_9# | PBP22992# | D | L 9; D 1 | CFEM domain | 9 |
| MbSSP23 | MBM_05535 | B2J93_9563 | PBP23932# | D | L 9; D 1 |  | 4 |
| MbSSP24 | MBM_03332 | B2J93_82# | PBP20623# | D | L 9; D 1 |  | 2 |
| MbSSP25 | MBM_07010 | B2J93_2777# | PBP18451# PBP18548# | L | L 8; D 1; S 1 |  | 4 |
| MbSSP26 | MBM_07169 | B2J93_5634 | PBP18100# | D | L 8; D 1; P 1 |  | 0 |
| MbSSP27 | MBM_03764 | B2J93_6494 | PBP16969 PBP20263 | L | L 8; D 1; E 1 | Transcription elongation factor Elf1 like | 8 |
| MbSSP28 | MBM_09206 | B2J93_9069# | PBP22198# | D | L 8; D 1; X 1 |  | 6 |
| MbSSP29 | MBM_08806 | B2J93_8466 | PBP15761 PBP28783 | D | L 8 |  | 3 |
| MbSSP30 | MBM_01661 | B2J93_6049 | PBP18459 | L | L 8; D 1; S 1 | Glycosyl hydrolase family 10 | 1 |
| MbSSP31 | MBM_05970 | B2J93_4363 | PBP27404 PBP15420 | L | L 7; D 3 | Pollen allergen | 6 |
| MbSSP32 | MBM_05395 | B2J93_4375 | PBP21215# PBP25185# | L | L 7; D 1; S 2 | Trm112p-like protein | 5 |
| MbSSP33 | MBM_05164 | B2J93_2124 | PBP18061# | D | L 6; D 1; E 3 |  | 3 |
| MbSSP34 | MBM_03877 | B2J93_4114# | PBP22972# PBP24234# | L | L 6; D 1 |  | 4 |
| MbSSP35 | MBM_03644 | B2J93_6889 | PBP16013# PBP20512# | D | L 6; D 3; E 1 | Cytidine and deoxycytidylate deaminase zinc-binding region | 9 |
| MbSSP36 | MBM_01252 | B2J93_7089# | PBP23642# | D | L 6; D 1; S 1; E 2 | Necrosis inducing protein (NPP1) | 4 |
| MbSSP37 | MBM_00405 | B2J93_8559# | PBP17512# | L | L 6; Bacteria 1; D 2; S 1 |  | 0 |
| MbSSP38 | MBM_08741 | B2J93_8565# | PBP17505# | L | L 5; D 5 |  | 1 |
| MbSSP39 | MBM_03198 | B2J93_726# | PBP21891# PBP16837# | L | L 5; D 3; S 2 | Cutinase | 4 |
| MbSSP40 | MBM_04851 | B2J93_7269# | PBP25837# | D | L 5; D 3; S 2 |  | 9 |
| MbSSP41 | MBM_09178 | B2J93_4420# | PBP21179# PBP16151# | D | L 5; D 3; S 2 |  | 11 |
| MbSSP42 | MBM_02256 | B2J93_2846 | PBP23299# | L | L 5; D 2; S 3 | 60s Acidic ribosomal protein | 0 |
| MbSSP43 | MBM_06072 | B2J93_3459# | PBP19799# | D | L 5; D 3; S 2 | Lytic polysaccharide mono-oxygenase, cellulose-degrading | 6 |
| MbSSP44 | MBM_00141 | B2J93_3620# | PBP26264# PBP21586# | D | L 5; E 3; D 1; Fis 1 | Pectate lyase | 10 |
| MbSSP45 | MBM_02478 | B2J93_5181# | PBP19448# PBP15643# | D | L 5; D 3; S 2 | Cerato-platanin | 4 |
| MbSSP46 | MBM_00109 | B2J93_3655# | PBP21613# PBP26280# | L | L 4; S 4; D 1 | Ser-Thr-rich glycosyl-phosphatidyl-inositol-anchored membrane family | 0 |
| MbSSP47 | MBM_07107 | B2J93_6460 | PBP16756# PBP17582# | L | L 4; D 6 |  | 2 |
| MbSSP48 | MBM_06694 | B2J93_7593# | PBP28906# | D | L 4; D 3; S 3 |  | 3 |
| MbSSP49 | MBM_06673 | B2J93_9431# | PBP26035# | D | L 4; D 4; S 2 |  | 5 |
| MbSSP50 | MBM_06658 | B2J93_5425 | PBP16331 | D | L 3; D 4; S 2; E 1 | Pectate lyase | 10 |
| MbSSP51 | MBM_04824 | B2J93_4624# | PBP22845# PBP24558# | D | L 3; D 1 |  | 6 |
| MbSSP52 | MBM_06081 | B2J93_3460 # | PBP19810# PBP15903# | D | L 3; D 3; S 4 | Peroxidase, family 2 | 2 |
| MbSSP53 | MBM_00286 | B2J93_1949# | PBP23319# PBP26284# | D | L 3; D 4; S 3 | Cutinase | 4 |
| MbSSP54 | MBM_01819 | B2J93_2761# | PBP18202# PBP23342# | D | L 3; D 6; P 1 |  | 8 |
| MbSSP55 | MBM_08351 | B2J93_8504# | PBP23677# PBP26346# | L | L 3; S1 |  | 0 |
| MbSSP56 | MBM_01161 | B2J93_2593# | PBP20618 | L | L 3; D 7 | Protein of unknown function (DUF1524) | 5 |
| MbSSP57 | MBM_01372 | B2J93_5149# | PBP18404# | L | L 2 |  | 3 |
| MbSSP58 | MBM_08471 | B2J93_6424# | PBP18894# PBP21706# | S | L 2; S 4; D 4 |  | 2 |
| MbSSP59 | MBM_05265 | B2J93_9345 | PBP15833 PBP16354 | D | L 2; D 9 | GDSL-like Lipase/Acylhydrolase family | 0 |
| MbSSP60 | MBM_08078 | B2J93_724# | PBP20730# | L | L 1; S 1; D 8 |  | 2 |
| MbSSP61 | MBM_01467 | B2J93_8304 | PBP15441# | L | L 1; D 1; E 3; S 4; P 1 | Polysaccharide deacetylase | 4 |
| MbSSP62 | MBM_08313 | B2J93_8986# | PBP25818# | D | L 1; D 7; S 1; E 1 |  | 0 |
| MbSSP63 | MBM_08651 | B2J93_499# | PBP26827# | S | S 4 | Complex I intermediate-associated protein 30 (CIA30) | 0 |
| MbSSP64 | MBM_02020 | B2J93_7020# | PBP21162# | D | D 1 |  | 6 |
| MbSSP65 | MBM_09600 | B2J93_8127 | PBP19660 | D | D 1; S 2 |  | 4 |
| MbSSP66 | MBM_06278 | B2J93_9614 | PBP22574# PBP25453# | O | O 1; Fis 1; D 2; S 1 |  | 1 |
| MbSSP67 | MBM_03679 | B2J93_6838# | PBP24165# PBP18492# | Unique | Unique |  | 0 |
| MbSSP68 | MBM_00728 | B2J93_988# | PBP16090# | Unique | Unique |  | 5 |
| MbSSP69 | MBM_00334 | B2J93_6574 | PBP18869 PBP26675 | Unique | Unique |  | 0 |
| MbSSP70 | MBM_04605 | B2J93_1689 | PBP21915# PBP21461# | Unique | Unique |  | 0 |
| MbSSP71 | MBM_06531 | B2J93_7297 |  | D | L 9; D 1 | CFEM domain | 8 |
| MbSSP72 | MBM_00547 | B2J93_8634# |  | D | L 9; D 1 | Isochorismatase family | 1 |
| MbSSP73 | MBM_07504 | B2J93_1751# |  | D | L 8; D 2 |  | 4 |
| MbSSP74 | MBM_09065 | B2J93_9104 |  | L | L 7; D 1; O 1; Fis 1 | CHRD domain | 3 |
| MbSSP75 | MBM_06672 | B2J93_2610 |  | L | L 7; E 1; S 1 |  | 4 |
| MbSSP76 | MBM_08007 | B2J93_5314# |  | L | L 6; D 1 |  | 5 |
| MbSSP77 | MBM_08179 | B2J93_3871# |  | D | L 5; D 5 | LysM domain | 4 |
| MbSSP78 | MBM_01882 | B2J93_5997# |  | D | L 5; D 3; S 2 |  | 0 |
| MbSSP79 | MBM_07831 | B2J93_1745 |  | L | L 3; D 2; O 2; S 2; E 1 | Cutinase | 7 |
| MbSSP80 | MBM_09719 | B2J93_7282 |  | D | L 2; S 1 |  | 2 |
| MbSSP81 | MBM_03077 | B2J93_8789 |  | D | L 1; D 1; S 8 |  | 4 |
| MbSSP82 | MBM_05156 | B2J93_2129 |  | D | D 1 |  | 3 |
| MbSSP83 | MBM_07301 | B2J93_878 |  | Unique | Unique |  | 4 |
| MbSSP84 | MBM_04139 | B2J93_1470# |  | Unique | Unique |  | 6 |
| MbSSP85 | MBM_08889 |  | PBP16574# | L | L 9; D 1 |  | 1 |
| Mbssp86 | MBM_01226 |  | PBP28634#  PBP22754# |  |  |  | 0 |
| MbSSP87 | MBM_03870 |  | PBP24268# | D | L 9; D 1 |  | 4 |
| MbSSP88 | MBM_05083 |  | PBP22233# | L | L 9; D 1 | Protein of unknown function (DUF1242) | 4 |
| MbSSP89 | MBM_07992 |  | PBP18625# PBP18967# | L | L 8; D 1; S 1 |  | 9 |
| MbSSP90 | MBM_02107 |  | PBP23189# PBP21256# | L | L 7; E 3 |  | 6 |
| MbSSP91 | MBM_06423 |  | PBP28423 PBP18128 | L | L 5; Fis 3 ; Slime mold 1; plant 1 | Chitin recognition protein | 12 |
| MbSSP92 | MBM_03589 |  | PBP27484# PBP15419# | D | L 2; D 6; S 2 |  | 4 |
| MbSSP93 | MBM_05906 |  | PBP22061# | D | D 1 |  | 0 |
| MbSSP94 | MBM_05246 |  | PBP27404 PBP27111 | Unique | Unique |  | 3 |
| MbSSP95 | MBM_07837* |  |  | L | L 9; D 1 |  | 4 |
| MbSSP96 | MBM_04662* |  |  | L | L 9 |  | 3 |
| MbSSP97 | MBM_02401* |  |  | L | L 9; B 1 | Cutinase | 4 |
| MbSSP98 | MBM_05476* |  |  | L | L 9; D 1 | Copper binding proteins, plastocyanin/azurin family | 4 |
| MbSSP99 | MBM_02928 |  |  | D | L 8; D 1; E 1 |  | 5 |
| MbSSP100 | MBM_04332* |  |  | L | L 7 |  | 11 |
| MbSSP101 | MBM_04516* |  |  | S | L 6; S 3; D 1 | CFEM domain | 8 |
| MbSSP102 | MBM_04770* |  |  | L | L 6; S 2 |  | 8 |
| MbSSP103 | MBM_07186* |  |  | L | L 6; D 3 | Fungal hydrophobin | 8 |
| MbSSP104 | MBM_03327 |  |  | L | L 5; D 5; |  | 4 |
| MbSSP105 | MBM_03844 |  |  | D | L 5; D 4; S 1 | Pectate lyase | 11 |
| MbSSP106 | MBM_04127* |  |  | L | L 5; S 5 |  | 3 |
| MbSSP107 | MBM_02646* |  |  | S | L 4; S 5; E 1 |  | 8 |
| MbSSP108 | MBM_08652 |  |  | L | L 4; D 2; S 3; Plant 1 |  | 4 |
| MbSSP109 | MBM_09396* |  |  | L | L 4 |  | 1 |
| MbSSP110 | MBM_01670* |  |  | D | L 4; D 1 |  | 8 |
| MbSSP111 | MBM_05202* |  |  | L | L 2; D 1 |  | 8 |
| MbSSP112 | MBM_03121* |  |  | Bacteria | L 2; Bacteria 1; S 3; D 4 |  | 14 |
| MbSSP113 | MBM_04107* |  |  | L | L 2; S 4; D 3 |  | 6 |
| MbSSP114 | MBM_07622* |  |  | L | L 2; S 2; B 1; D 2 |  | 2 |
| MbSSP115 | MBM_07385* |  |  | L | L 2; A 2 |  | 7 |
| MbSSP116 | MBM_02229* |  |  | L | L 1 |  | 9 |
| MbSSP117 | MBM_01389* |  |  | L | L 1; D 9 |  | 2 |
| MbSSP118 | MBM_00672* |  |  | D | L 1; D 7; E 1; S 1 | SnoaL-like domain | 2 |
| MbSSP119 | MBM_05341* |  |  | L | L 1; D 1 |  | 6 |
| MbSSP120 | MBM_00667* |  |  | S | S 3; D 4 | LysM domain | 6 |
| MbSSP121 | MBM_00892* |  |  | S | S 3; D 4 | LysM domain | 6 |
| MbSSP122 | MBM_00944* |  |  | S | S 3; D 5 | LysM domain | 6 |
| MbSSP123 | MBM_05794* |  |  | S | S 4; D 3 | LysM domain | 6 |
| MbSSP124 | MBM_06094* |  |  | S | S 3; D 5 | LysM domain | 6 |
| MbSSP125 | MBM_09190* |  |  | S | S 3; D 5 | LysM domain | 6 |
| MbSSP126 | MBM_01522* |  |  | S | S 2; D 5 | LysM domain | 6 |
| MbSSP127 | MBM_08215* |  |  | S | S 2; D 4 | LysM domain | 6 |
| MbSSP128 | MBM_07673* |  |  | S | S 3; D 4 | LysM domain | 6 |
| MbSSP129 | MBM_08223* |  |  | D | D 4; S 3 | LysM domain | 6 |
| MbSSP130 | MBM_08224* |  |  | S | S 2; D 4 | LysM domain | 6 |
| MbSSP131 | MBM_09419* |  |  | S | S 2; D 4 | LysM domain | 6 |
| MbSSP132 | MBM_09513* |  |  | S | S 3; D 5 | LysM domain | 6 |
| MbSSP133 | MBM_09653* |  |  | S | S 3; D 3 | LysM domain | 6 |
| MbSSP134 | MBM_03835* |  |  | S | S 2; D 4 | LysM domain | 6 |
| MbSSP135 | MBM_03863* |  |  | S | S 3; D 1 | LysM domain | 6 |
| MbSSP136 | MBM_00291* |  |  | S | S 2; D 5 | LysM domain | 6 |
| MbSSP137 | MBM_06470* |  |  | S | S 3; D 5 | LysM domain | 6 |
| MbSSP138 | MBM_05221* |  |  | S | S 2; D 2 | LysM domain | 6 |
| MbSSP139 | MBM_05370* |  |  | S | S 3; D 3 | LysM domain | 7 |
| MbSSP140 | MBM_07277* |  |  | S | S 3; S 5 | LysM domain | 6 |
| MbSSP141 | MBM_01073* |  |  | S | S 3; D 2 | LysM domain | 6 |
| MbSSP142 | MBM_03066* |  |  | S | S 3; D 5 | LysM domain | 6 |
| MbSSP143 | MBM_09952* |  |  | S | S 3; D 3 | LysM domain | 7 |
| MbSSP144 | MBM_03916* |  |  | S | S 1 | IGY | 2 |
| MbSSP145 | MBM_07348* |  |  | S | S 1 | IGY | 2 |
| MbSSP146 | MBM_07373* |  |  | S | S 1 | IGY | 2 |
| MbSSP147 | MBM_01039* |  |  | S | S 1 | IGY | 4 |
| MbSSP148 | MBM_01069* |  |  | S | S 1 | IGY | 3 |
| MbSSP149 | MBM_06106* |  |  | S | S 1 | IGY | 1 |
| MbSSP150 | MBM_06114* |  |  | S | S 1 | IGY | 3 |
| MbSSP151 | MBM_06513* |  |  | S | S 1 | IGY | 2 |
| MbSSP152 | MBM_06516* |  |  | S | S 1 | IGY | 2 |
| MbSSP153 | MBM_06517* |  |  | S | S 1 | IGY | 2 |
| MbSSP154 | MBM_06608* |  |  | S | S 1 | IGY | 3 |
| MbSSP155 | MBM_06410* |  |  | S | S 1 | IGY | 3 |
| MbSSP156 | MBM_06288* |  |  | S | S 1 | IGY | 3 |
| MbSSP157 | MBM_07463* |  |  | S | S1 | IGY | 2 |
| MbSSP158 | MBM_09239* |  |  | S | S 1 | IGY | 3 |
| MbSSP159 | MBM_01655* |  |  | S | S 1 | IGY | 3 |
| MbSSP160 | MBM_03635* |  |  | S | S 1 | IGY | 2 |
| MbSSP161 | MBM_03774* |  |  | S | S 1 | IGY | 3 |
| MbSSP162 | MBM_03043* |  |  | S | S 1 | IGY | 2 |
| MbSSP163 | MBM_03065* |  |  | S | S 1 | IGY | 2 |
| MbSSP164 | MBM_09514* |  |  | S | S 1 | IGY | 3 |
| MbSSP165 | MBM_09523* |  |  | S | S 1 | IGY | 3 |
| MbSSP166 | MBM_02300* |  |  | S | S 1 | IGY | 5 |
| MbSSP167 | MBM_02459* |  |  | S | S 1 | IGY | 2 |
| MbSSP168 | MBM_02634* |  |  | S | S 1 | IGY | 3 |
| MbSSP169 | MBM_02762* |  |  | S | S 1 | IGY | 2 |
| MbSSP170 | MBM_02801* |  |  | S | S 1 | IGY | 2 |
| MbSSP171 | MBM_02933* |  |  | S | S 1 | IGY | 3 |
| MbSSP172 | MBM_02979* |  |  | S | S 1 | IGY | 2 |
| MbSSP173 | MBM_03125* |  |  | S | S 1 | IGY | 3 |
| MbSSP174 | MBM_09467* |  |  | S | S 1 | IGY | 3 |
| MbSSP175 | MBM_09484* |  |  | S | S 1 | IGY | 3 |
| MbSSP176 | MBM_09485* |  |  | S | S 1 | IGY | 3 |
| MbSSP177 | MBM_01657* |  |  | S | S 1 | IGY | 3 |
| MbSSP178 | MBM_01673* |  |  | S | S 1 | IGY | 3 |
| MbSSP179 | MBM_01935* |  |  | S | S 1 | IGY | 4 |
| MbSSP180 | MBM_01967* |  |  | S | S 1 | IGY | 2 |
| MbSSP181 | MBM_02008* |  |  | S | S 1 | IGY | 2 |
| MbSSP182 | MBM_02064* |  |  | S | S 1 | IGY | 2 |
| MbSSP183 | MBM_08562* |  |  | S | S 1 | IGY | 3 |
| MbSSP184 | MBM_08603* |  |  | S | S 1 | IGY | 3 |
| MbSSP185 | MBM_08781* |  |  | S | S 1 | IGY | 5 |
| MbSSP186 | MBM_08804* |  |  | S | S 1 | IGY | 4 |
| MbSSP187 | MBM_08817* |  |  | S | S 1 | IGY | 3 |
| MbSSP188 | MBM_08751* |  |  | S | S 1 | IGY | 3 |
| MbSSP189 | MBM_08893* |  |  | S | S 1 | IGY | 2 |
| MbSSP190 | MBM_06164* |  |  | S | S 1 | IGY | 4 |
| MbSSP191 | MBM_06457* |  |  | S | S 1 | IGY | 2 |
| MbSSP192 | MBM_06553* |  |  | S | S 1 | IGY | 4 |
| MbSSP193 | MBM_06742* |  |  | S | S 1 | IGY | 3 |
| MbSSP194 | MBM_07030* |  |  | S | S 1 | IGY | 3 |
| MbSSP195 | MBM_01284* |  |  | S | S 1 | IGY | 2 |
| MbSSP196 | MBM_01411* |  |  | S | S 1 | IGY | 3 |
| MbSSP197 | MBM_05867* |  |  | S | S 1 | IGY | 4 |
| MbSSP198 | MBM_06078* |  |  | S | S 1 | IGY | 2 |
| MbSSP199 | MBM_04990* |  |  | S | S 1 | IGY | 6 |
| MbSSP200 | MBM_05036* |  |  | S | S 1 | IGY | 2 |
| MbSSP201 | MBM_05180* |  |  | S | S 1 | IGY | 2 |
| MbSSP202 | MBM_05309* |  |  | S | S 1 | IGY | 2 |
| MbSSP203 | MBM_05496* |  |  | S | S 1 | IGY | 3 |
| MbSSP204 | MBM_05353* |  |  | S | S 1; E 2 | IGY | 3 |
| MbSSP205 | MBM_05805* |  |  | S | S 1 | IGY | 2 |
| MbSSP206 | MBM_05835* |  |  | S | S 1 | IGY | 2 |
| MbSSP207 | MBM_06397* |  |  | S | S 1 | IGY | 2 |
| MbSSP208 | MBM_06530* |  |  | S | S 1 | IGY | 3 |
| MbSSP209 | MBM_06972* |  |  | S | S 1 | IGY | 2 |
| MbSSP210 | MBM_07003* |  |  | S | S 1 | IGY | 2 |
| MbSSP211 | MBM_07127* |  |  | S | S 1 | IGY | 3 |
| MbSSP212 | MBM_07626* |  |  | S | S 1 | IGY | 2 |
| MbSSP213 | MBM_01554* |  |  | S | S 2; E 1 | IGY | 2 |
| MbSSP214 | MBM_01285* |  |  | S | S 1 | IGY | 2 |
| MbSSP215 | MBM_08812* |  |  | S | S 1 | IGY | 2 |
| MbSSP216 | MBM_01666* |  |  | S | S 1 | IGY | 2 |
| MbSSP217 | MBM_01885* |  |  | S | S 1 | IGY | 2 |
| MbSSP218 | MBM_02783* |  |  | S | S 2; E 1 | IGY | 2 |
| MbSSP219 | MBM_02784* |  |  | S | S 1 | IGY | 2 |
| MbSSP220 | MBM_02930* |  |  | S | S 1 | IGY | 2 |
| MbSSP221 | MBM_02984* |  |  | S | S 1 | IGY | 2 |
| MbSSP222 | MBM_00192* |  |  | S | S 2 | IGY | 2 |
| MbSSP223 | MBM_00459* |  |  | S | S 1 | IGY | 2 |
| MbSSP224 | MBM_00874* |  |  | S | S 1 | IGY | 2 |
| MbSSP225 | MBM_04645* |  |  | S | S 1 | IGY | 2 |
| MbSSP226 | MBM_00662* |  |  | S | S 1 | IGY | 3 |
| MbSSP227 | MBM_00415* |  |  | S | S 1 | IGY | 2 |
| MbSSP228 | MBM_04758* |  |  | S | S 1 | IGY | 2 |
| MbSSP229 | MBM_04664* |  |  | S | S 1 | IGY | 2 |
| MbSSP230 | MBM_00946* |  |  | S | S 1 | IGY | 2 |
| MbSSP231 | MBM_05855* |  |  | S | S 2; D 3 |  | 6 |
| MbSSP232 | MBM_00913* |  |  | D | D 1 |  | 5 |
| MbSSP233 | MBM_04525* |  |  | S | S 3; D 2 |  | 6 |
| MbSSP234 | MBM_05696* |  |  | S | S 3 |  | 8 |
| MbSSP235 | MBM_09373* |  |  | S | S 2; D 5 |  | 6 |
| MbSSP236 | MBM_08294* |  |  | S | S 1 |  | 6 |
| MbSSP237 | MBM_09163* |  |  | S | S 5; D 5 |  | 8 |
| MbSSP238 | MBM_02395* |  |  | S | S 3; D 3 |  | 3 |
| MbSSP239 | MBM_04282* |  |  | S | S 3; D 1 |  | 6 |
| MbSSP240 | MBM_06820* |  |  | D | D 3; S 2 |  | 6 |
| MbSSP241 | MBM_06455* |  |  | Unique | Unique |  | 4 |
| MbSSP242 | MBM_00970* |  |  | Unique | Unique |  | 6 |
| MbSSP243 | MBM_05528* |  |  | Unique | Unique |  | 6 |
| MbSSP244 | MBM_00980* |  |  | Unique | Unique |  | 4 |
| MbSSP245 | MBM_01089* |  |  | Unique | Unique |  | 8 |
| MbSSP246 | MBM_01510* |  |  | Unique | Unique |  | 4 |
| MbSSP247 | MBM_07182* |  |  | Unique | Unique |  | 0 |
| MbSSP248 | MBM_01605* |  |  | Unique | Unique |  | 6 |
| MbSSP249 | MBM_01758* |  |  | Unique | Unique |  | 7 |
| MbSSP250 | MBM_07745* |  |  | Unique | Unique |  | 1 |
| MbSSP251 | MBM_07836* |  |  | Unique | Unique |  | 7 |
| MbSSP252 | MBM_08546* |  |  | Unique | Unique |  | 6 |
| MbSSP253 | MBM_02629* |  |  | Unique | Unique |  | 0 |
| MbSSP254 | MBM_04915* |  |  | Unique | Unique |  | 6 |
| MbSSP255 | MBM_03364* |  |  | Unique | Unique |  | 4 |
| MbSSP256 | MBM_04012* |  |  | Unique | Unique |  | 3 |
| MbSSP257 | MBM_00338* |  |  | Unique | Unique |  | 7 |
| MbSSP258 | MBM_00534* |  |  | Unique | Unique |  | 3 |
| MbSSP259 | MBM_02822* |  |  | Unique | Unique |  | 2 |
| MbSSP260 | MBM_00918* |  |  | Unique | Unique |  | 2 |
| MbSSP261 | MBM_04737* |  |  | Unique | Unique |  | 6 |
| MbSSP262 | MBM_04857* |  |  | Unique | Unique |  | 3 |
| MbSSP263 | MBM_04899* |  |  | Unique | Unique |  | 0 |
| MbSSP264 | MBM_05120* |  |  | Unique | Unique |  | 6 |
| MbSSP265 | MBM_06256* |  |  | Unique | Unique |  | 6 |
| MbSSP266 | MBM_01278* |  |  | Unique | Unique |  | 6 |
| MbSSP267 | MBM_01549* |  |  | Unique | Unique |  | 5 |
| MbSSP268 | MBM_08024* |  |  | Unique | Unique |  | 5 |
| MbSSP269 | MBM_01375* |  |  | Unique | Unique |  | 7 |
| MbSSP270 | MBM_08328* |  |  | Unique | Unique |  | 3 |
| MbSSP271 | MBM_08384* |  |  | Unique | Unique |  | 1 |
| MbSSP272 | MBM_08427* |  |  | Unique | Unique |  | 0 |
| MbSSP273 | MBM_08482* |  |  | Unique | Unique |  | 8 |
| MbSSP274 | MBM_08775* |  |  | Unique | Unique |  | 6 |
| MbSSP275 | MBM_08772* |  |  | Unique | Unique |  | 6 |
| MbSSP276 | MBM_08216* |  |  | Unique | Unique |  | 2 |
| MbSSP277 | MBM_09208* |  |  | Unique | Unique |  | 3 |
| MbSSP278 | MBM_09209* |  |  | Unique | Unique |  | 6 |
| MbSSP279 | MBM_09244* |  |  | Unique | Unique |  | 8 |
| MbSSP280 | MBM_08818* |  |  | Unique | Unique |  | 4 |
| MbSSP281 | MBM_01588* |  |  | Unique | Unique |  | 6 |
| MbSSP282 | MBM_02130* |  |  | Unique | Unique |  | 6 |
| MbSSP283 | MBM_09393* |  |  | Unique | Unique |  | 3 |
| MbSSP284 | MBM_09418* |  |  | Unique | Unique |  | 5 |
| MbSSP285 | MBM_08989* |  |  | Unique | Unique |  | 1 |
| MbSSP286 | MBM_09067* |  |  | Unique | Unique |  | 2 |
| MbSSP287 | MBM_09081* |  |  | Unique | Unique |  | 6 |
| MbSSP288 | MBM_09420* |  |  | Unique | Unique |  | 2 |
| MbSSP289 | MBM_09428* |  |  | Unique | Unique |  | 8 |
| MbSSP290 | MBM_09490* |  |  | Unique | Unique |  | 6 |
| MbSSP291 | MBM_09570* |  |  | Unique | Unique |  | 5 |
| MbSSP292 | MBM_09610* |  |  | Unique | Unique |  | 6 |
| MbSSP293 | MBM_09622* |  |  | Unique | Unique |  | 2 |
| MbSSP294 | MBM_02282* |  |  | Unique | Unique |  | 6 |
| MbSSP295 | MBM_02283* |  |  | Unique | Unique |  | 6 |
| MbSSP296 | MBM_02429* |  |  | Unique | Unique |  | 6 |
| MbSSP297 | MBM_02640* |  |  | Unique | Unique |  | 3 |
| MbSSP298 | MBM_02674* |  |  | Unique | Unique |  | 0 |
| MbSSP299 | MBM_02980* |  |  | Unique | Unique |  | 0 |
| MbSSP300 | MBM_02989* |  |  | Unique | Unique |  | 6 |
| MbSSP301 | MBM_10022* |  |  | Unique | Unique |  | 7 |
| MbSSP302 | MBM_10023* |  |  | Unique | Unique |  | 7 |
| MbSSP303 | MBM_03470* |  |  | Unique | Unique |  | 4 |
| MbSSP304 | MBM_03560* |  |  | Unique | Unique |  | 6 |
| MbSSP305 | MBM_03807* |  |  | Unique | Unique |  | 7 |
| MbSSP306 | MBM_05139* |  |  | Unique | Unique |  | 3 |
| MbSSP307 | MBM_05145* |  |  | Unique | Unique |  | 9 |
| MbSSP308 | MBM_05217* |  |  | Unique | Unique |  | 6 |
| MbSSP309 | MBM_05219* |  |  | Unique | Unique |  | 6 |
| MbSSP310 | MBM_06124* |  |  | Unique | Unique |  | 2 |
| MbSSP311 | MBM_06340* |  |  | Unique | Unique |  | 0 |
| MbSSP312 | MBM_06354* |  |  | Unique | Unique |  | 0 |
| MbSSP313 | MBM_06421* |  |  | Unique | Unique |  | 8 |
| MbSSP314 | MBM_06557* |  |  | Unique | Unique |  | 6 |
| MbSSP315 | MBM_06622* |  |  | Unique | Unique |  | 3 |
| MbSSP316 | MBM_06629* |  |  | Unique | Unique |  | 6 |
| MbSSP317 | MBM_06744* |  |  | Unique | Unique |  | 0 |
| MbSSP318 | MBM_06785* |  |  | Unique | Unique | LysM domain | 6 |
| MbSSP319 | MBM_07491* |  |  | Unique | Unique |  | 3 |
| MbSSP320 | MBM_01033* |  |  | Unique | Unique |  | 8 |
| MbSSP321 | MBM_01058* |  |  | Unique | Unique |  | 6 |
| MbSSP322 | MBM_00981* |  |  | Unique | Unique |  | 6 |
| MbSSP323 | MBM_05369* |  |  | Unique | Unique |  | 6 |
| MbSSP324 | MBM_04330* |  |  | Unique | Unique |  | 6 |

a The hits from one genus were counted only once. E, Eurotiomycetes; S, Sordariomycetes; L, Leotiomycetes; D, Dothideomycetes; X, Xylonomycetes; P, Pezizomycotina incertae sedis, C, Lecanoromycetes, O, Orbiliomycetes, A, Saccharomycetes; F, Schizosaccharomycetes; G, Basidiobolomycetes; B, Basidiomycota; Fis, Fungi incertae sedis; Slime mold, Amoebozoa Eumycetozoa; Animal, Metazoa; Plant, Viridiplantae magnoliopsida

Yellow grid, orthologs confirmed by the best-reciprocal-hit Blast

b Protein domain was predicted by Pfam (<http://pfam.xfam.org/>) and blastp in NCBI NR database.

*Species-specific SSPs

# SSPs in *M. coronaria* and *M. rosae*.
