# Supplementary material for: Draft genome sequence of Marssonina coronaria, causal agent of apple blotch, and comparisons with the Marssonina brunnea and Marssonina rosae genomes
Source: PLoS One. 2021 Feb 5;16(2):e0246666. doi: 10.1371/journal.pone.0246666 (PMC7864672; doi:10.1371/journal.pone.0246666)
Supplement: S10 Table — (DOCX) [file pone.0246666.s011.docx]

**S10 Table.** The summary of the small secreted proteins of *Marssonina rosae*

| Gene name | *Marssonina rosae* | Nucleotide identity^a^ | *Marssonina*  *brunnea* | *Marssonina coronaria* | Taxonomy of the first hit^b^ | Taxonomy of the top10 best hits^b^ | Description^c^ | Cysteine residue |
| --- | --- | --- | --- | --- | --- | --- | --- | --- |
| DrSSP1 | PBP17192 |  | MBM_00349 | B2J93_6592 | L | L 10 | DNA/RNA non-specific endonuclease | 2 |
| DrSSP2 | PBP27545 PBP26758 | 95.3% | MBM_00915# | B2J93_6771# | L | L 10 | FKBP-type peptidyl-prolyl cis-trans isomerase | 2  2 |
| DrSSP3 | PBP28729 PBP22726 | 89.3% | MBM_01208# | B2J93_3113 | L | L 10 |  | 7  3 |
| DrSSP4 | PBP21072 |  | MBM_01747 | B2J93_7466 | L | L 10 | Cutinase | 4 |
| DrSSP5 | PBP25519 PBP21457 | 95% | MBM_02168# | B2J93_8195# | L | L 10 | Killer toxin-resistance protein 1 | 0  0 |
| DrSSP6 | PBP28164 |  | MBM_02262 | B2J93_2545# | L | L 10 |  | 2 |
| DrSSP7 | PBP27983 |  | MBM_02294 | B2J93_2571 | L | L 10 |  | 0 |
| DrSSP8 | PBP25498 |  | MBM_04245 | B2J93_7289# | L | L 10 |  | 4 |
| DrSSP9 | PBP25853 |  | MBM_04864 | B2J93_7259# | L | L 10 | Cysteine-rich secretory protein family | 4 |
| DrSSP10 | PBP18814 |  | MBM_03438# | B2J93_7915 | L | L 10 |  | 1 |
| DrSSP11 | PBP22229 PBP21731 | 94.1% | MBM_05034# | B2J93_6784# | L | L 10 | emp24/gp25L/p24 family/GOLD | 3  3 |
| DrSSP12 | PBP22266 PBP21744 | 77.7% | MBM_05118# | B2J93_5326# | L | L 10 | CFEM domain | 8  8 |
| DrSSP13 | PBP23932 |  | MBM_05535# | B2J93_9563 | L | L 10 |  | 5 |
| DrSSP14 | PBP23944 |  | MBM_05600 | B2J93_372 | L | L 10 | LicD family | 1 |
| DrSSP15 | PBP21099 |  | MBM_05647 | B2J93_3982 | L | L 10 | Phosphatidylethanolamine-binding protein | 0 |
| DrSSP16 | PBP17786 |  | MBM_05286 | B2J93_1852 | L | L 10 | Glycosyl hydrolase catalytic core | 0 |
| DrSSP17 | PBP25185 PBP21215 | 90.4% | MBM_05395# | B2J93_4375 | L | L 10 | Trm112p-like protein | 5  5 |
| DrSSP18 | PBP27322 PBP26952 | 92.4% | MBM_05824# | B2J93_229# | L | L 10 |  | 10  10 |
| DrSSP19 | PBP27039 PBP20673 | 93.1% | MBM_05877# | B2J93_2694 | L | L 10 | emp24/gp25L/p24 family/GOLD | 2  2 |
| DrSSP20 | PBP22992 |  | MBM_06307# | B2J93_9# | L | L 10 | CFEM domain | 8 |
| DrSSP21 | PBP28259 PBP24651 | 93.9% | MBM_06958# | B2J93_888# | L | L 10 |  | 3  3 |
| DrSSP22 | PBP18548 PBP18451 | 91.7% | MBM_07010# | B2J93_2777# | L | L 10 | Plastocyanin-like domain | 3  3 |
| DrSSP23 | PBP28293 |  | MBM_07274 | B2J93_5485 | L | L 10 | Ferritin-like domain | 3 |
| DrSSP24 | PBP18248 |  | MBM_07698 | B2J93_6147 | L | L 10 |  | 5 |
| DrSSP25 | PBP25111 PBP20890 | 91.4% | MBM_07761# | B2J93_1682# | L | L 10 | Egh16-like virulence factor | 6  6 |
| DrSSP26 | PBP19028 PBP18087 | 91.0% | MBM_07937# | B2J93_5650# | L | L 10 | Protein of unknown function (DUF3455) | 4  4 |
| DrSSP27 | PBP21698 PBP15393 | 78.9% | MBM_08054 | B2J93_3912 | L | L 10 | CHD5-like protein | 2  2 |
| DrSSP28 | PBP25090 |  | MBM_08076# | B2J93_6267 | L | L 10 |  | 5 |
| DrSSP29 | PBP16773 |  | MBM_08162# | B2J93_5015# | L | L 10 |  | 5 |
| DrSSP30 | PBP21281 |  | MBM_09097 | B2J93_4299 | L | L 10 |  | 5 |
| DrSSP31 | PBP23382 |  | MBM_08458 | B2J93_8055# | L | L 10 | Ubiquitin 3 binding protein But2 C-terminal domain | 4 |
| DrSSP32 | PBP28277 PBP27202 | 91.2% | MBM_08331 | B2J93_846# | L | L 10 |  | 4  5 |
| DrSSP33 | PBP22198 |  | MBM_09206# | B2J93_9069# | L | L 10 |  | 4 |
| DrSSP34 | PBP16508 |  | MBM_09400# | B2J93_7651# | L | L 10 |  | 2 |
| DrSSP35 | PBP25684 |  | MBM_09528 | B2J93_3456 | L | L 10 |  | 1 |
| DrSSP36 | PBP25685 PBP23248 | 90.8% | MBM_09529# | B2J93_3084 | L | L 10 |  | 1  1 |
| DrSSP37 | PBP17512 |  | MBM_00405# | B2J93_8559# | D | L 9; Bacteria 1 |  | 0 |
| DrSSP38 | PBP17212 |  | MBM_01783 | B2J93_1978# | L | L 9; E 1 |  | 4 |
| DrSSP39 | PBP19448 PBP15643 | 89.7% | MBM_02478# | B2J93_5181# | L | L 9; S 1 | Cerato-platanin | 4  4 |
| DrSSP40 | PBP21211 |  | MBM_05401 | B2J93_4381 | L | L 9; S 1 |  | 2 |
| DrSSP41 | PBP19799 |  | MBM_06072# | B2J93_3459# | L | L 9; D 1 | Lytic polysaccharide mono-oxygenase, cellulose-degrading | 6 |
| DrSSP42 | PBP18100 |  | MBM_07169# | B2J93_5634 | L | L 9; P 1 |  | 0 |
| DrSSP43 | PBP21179 PBP16151 | 88.2% | MBM_09178# | B2J93_4420# | D | L 9; D 1 |  | 3  3 |
| DrSSP44 | PBP21232 |  | MBM_09891 | B2J93_5139 | L | L 8; D 1; S 1 | Glycosyl hydrolase family 61 | 5 |
| DrSSP45 | PBP18404 |  | MBM_01372# | B2J93_5149# | L | L 8; D 2 |  | 9 |
| DrSSP46 | PBP16439 |  | MBM_01523 | B2J93_789 | L | L 8; E 1; D 2 | Lytic polysaccharide mono-oxygenase, cellulose-degrading | 3 |
| DrSSP47 | PBP28139 |  | MBM_02028 | B2J93_3968# | L | L 8; B 1; S 1 |  | 2 |
| DrSSP48 | PBP28323 PBP18523 | 76.3% | MBM_02513 | B2J93_5511# | L | L 8; S 2 | Ser-Thr-rich glycosyl-phosphatidyl-inositol-anchored membrane family | 0  0 |
| DrSSP49 | PBP20623 |  | MBM_03332# | B2J93_82# | L | L 8; S 2 |  | 3 |
| DrSSP50 | PBP18109 |  | MBM_03813 | B2J93_2606# | L | L 8; D 2 |  | 7 |
| DrSSP51 | PBP25837 |  | MBM_04851# | B2J93_7269# | L | L 8; S 1 |  | 13 |
| DrSSP52 | PBP19571 |  | MBM_06153# | B2J93_7733# | L | L 8 |  | 4 |
| DrSSP53 | PBP26035 |  | MBM_06673# | B2J93_9431# | L | L 8; D 1; S 1 |  | 4 |
| DrSSP54 | PBP18905 PBP18123 | 93.8% | MBM_07038 | B2J93_2613 | L | L 8; E 2 | Glutathione S-transferase, N-terminal domain | 2  2 |
| DrSSP55 | PBP25818 |  | MBM_08313# | B2J93_8986# | L | L 8; O 1; D 1 |  | 0 |
| DrSSP56 | PBP17000 PBP16799 | 49.7% | MBM_04999 | B2J93_2387 | S | L 7; S 2; E 1 | CFEM domain | 6  8 |
| DrSSP57 | PBP18061 |  | MBM_05164# | B2J93_2124 | L | L 7; S 2; E 1 |  | 3 |
| DrSSP58 | PBP26280 PBP21613 | 84.9% | MBM_00109# | B2J93_3655# | L | L 7; S 3 | Ser-Thr-rich glycosyl-phosphatidyl-inositol-anchored membrane family | 1  1 |
| DrSSP59 | PBP26264 PBP21586 | 91.9% | MBM_00141# | B2J93_3620# | L | L 7; Fis 1; S 1; D 1 | Pectate lyase | 10  10 |
| DrSSP60 | PBP27939 |  | MBM_01170 | B2J93_8188 | L | L 7 |  | 4 |
| DrSSP61 | PBP20512 PBP16013 | 86.1% | MBM_03644# | B2J93_6889 | L | L 7; D 2; E 1 | Cytidine and deoxycytidylate deaminase zinc-binding region | 2  9 |
| DrSSP62 | PBP24234 PBP22972 | 93.6% | MBM_03877# | B2J93_4114# | L | L 7 |  | 5  5 |
| DrSSP63 | PBP28433 PBP16418 | 88.5% | MBM_00538 | B2J93_8622 | L | L 6; S 3; O 1 |  | 0  0 |
| DrSSP64 | PBP23642 |  | MBM_01252# | B2J93_7089# | L | L 6; S 1; D 1; E 2 |  | 4 |
| DrSSP65 | PBP23342 PBP18202 | 80.7% | MBM_01819# | B2J93_2761# | L | L 6; P 1; S 1; D 2 |  | 8  8 |
| DrSSP66 | PBP23299 |  | MBM_02256# | B2J93_2846 | L | L 6; S 3; D 1 | 60s Acidic ribosomal protein | 0 |
| DrSSP67 | PBP15842 |  | MBM_04125 | B2J93_5047 | L | L 6; S 3; |  | 1 |
| DrSSP68 | PBP28906 |  | MBM_06694# | B2J93_7593# | L | L 6; D 4 |  | 4 |
| DrSSP69 | PBP17582 PBP16756 | 92.8% | MBM_07107# | B2J93_6460 | L | L 6; D 4 |  | 2  2 |
| DrSSP70 | PBP15418 |  | MBM_08888 | B2J93_2310 | L | L 5; O 1; D 4 | Eukaryotic-type carbonic anhydrase | 3 |
| DrSSP71 | PBP21891 PBP16837 | 91.4% | MBM_03198# | B2J93_726# | L | L 5; S 3; D 2 | Cutinase | 4  5 |
| DrSSP72 | PBP28481 PBP15802 | 89.1% | MBM_06273 | B2J93_6070# | L | L 5 | CFEM domain | 8  8 |
| DrSSP73 | PBP24581 |  | MBM_06721 | B2J93_7810 | L | L 5; E 5 | Common central domain of tyrosinase | 6 |
| DrSSP74 | PBP19810 PBP15903 | 66.7% | MBM_06081# | B2J93_3460# | L | L 4; D 1; S 5 | Peroxidase, family 2 | 3  3 |
| DrSSP75 | PBP25205 PBP15383 | 85.7% | MBM_04229 | B2J93_1745 | L | L 4; O 2; S 2; D 2 | Cutinase | 4  2 |
| DrSSP76 | PBP16183 |  | MBM_05777 | B2J93_4249# | L | L 4 |  | 3 |
| DrSSP77 | PBP26284 PBP23319 | 80.9% | MBM_00286# | B2J93_1949# | L | L 3; S 3; D 4 | Cutinase | 5  4 |
| DrSSP78 | PBP24296 |  | MBM_02474 | B2J93_3800 | L | L 3; O 2; D 3; A 1; S 1 | Pectate lyase | 4 |
| DrSSP79 | PBP26346 PBP23677 | 79.8% | MBM_08351# | B2J93_8504# | L | L 3; S 1 |  | 0  0 |
| DrSSP80 | PBP23475 |  | MBM_07556 | B2J93_6405 | D | L 3; D 2; S 5 |  | 2 |
| DrSSP81 | PBP25132 PBP21196 | 91.6% | MBM_07840 | B2J93_1755 | L | L 3; D 4; S 3 |  | 11  11 |
| DrSSP82 | PBP15441 |  | MBM_01467# | B2J93_8304 | L | L 2; S 6; P 1; D 1 | Polysaccharide deacetylase | 5 |
| DrSSP83 | PBP20730 |  | MBM_08078# | B2J93_724# | L | L 2; D 7; S 1 |  | 3 |
| DrSSP84 | PBP26827 |  | MBM_08651# | B2J93_499# | S | L 2; S 5; M 1 | Complex I intermediate-associated protein 30 (CIA30) | 4 |
| DrSSP85 | PBP17505 |  | MBM_08741# | B2J93_8565# | L | L 2; D 8 |  | 0 |
| DrSSP86 | PBP21706 PBP18894 | 77.3% | MBM_08471# | B2J93_6424# | S | L 1; S 5;D 4 |  | 2  3 |
| DrSSP87 | PBP15478 |  | MBM_08896 | B2J93_8314 | L | L 1 | Thioredoxin-like domain | 4 |
| DrSSP88 | PBP18918 |  | MBM_09907 | B2J93_5291# | L | L 1; D 6; S 3 |  | 4 |
| DrSSP89 | PBP21162 |  | MBM_02020# | B2J93_7020# | L | L 1 |  | 7 |
| DrSSP90 | PBP24165 PBP18492 | 84.6% | MBM_03679# | B2J93_6838# | L | L 1 |  | 1  2 |
| DrSSP91 | PBP22845 PBP24558 | 86.3% | MBM_04824# | B2J93_4624# | L | L 1 |  | 6  4 |
| DrSSP92 | PBP21482 |  | MBM_04551 | B2J93_5956 | S | L 1; S 3; D 6 | Glycosyl hydrolase family 61 | 4 |
| DrSSP93 | PBP25672 PBP19957 | 93.4% | MBM_05194 | B2J93_1563# | L | L 1 |  | 3  3 |
| DrSSP94 | PBP25453 PBP22574 | 89.8% | MBM_06278# | B2J93_9614 | Fis | L 1; Fis 1;O 1; D 1;S 1 |  | 0  0 |
| DrSSP95 | PBP25906 |  | MBM_09743 | B2J93_7106 | Unique | Unique |  | 7 |
| DrSSP96 | PBP16090 |  | MBM_00728# | B2J93_988# | Unique | Unique |  | 5 |
| DrSSP97 | PBP21915 PBP21461 | 88% | MBM_04605# | B2J93_5908 | Unique | Unique |  | 0  0 |
| DrSSP98 | PBP28634 PBP22754 | 72.2% | MBM_01226# |  | L | L 10 |  | 2  0 |
| DrSSP99 | PBP16574 |  | MBM_08889# |  | L | L 10 |  | 2 |
| DrSSP100 | PBP18967 PBP18625 | 67% | MBM_07992# |  | L | L 10 |  | 6  8 |
| DrSSP101 | PBP22233 |  | MBM_05083# |  | L | L 10 | Protein of unknown function (DUF1242) | 5 |
| DrSSP102 | PBP16729 |  | MBM_06607 |  | L | L 10 | ML domain | 4 |
| DrSSP103 | PBP23189 PBP21256 | 91.9% | MBM_02107# |  | L | L 8; E 2 |  | 6  6 |
| DrSSP104 | PBP24268 |  | MBM_03870# |  | L | L 6; S 4 |  | 4 |
| DrSSP105 | PBP22061 |  | MBM_05906# |  | L | L 6 |  | 0 |
| DrSSP106 | PBP18438 |  | MBM_08868 |  | D | L 1; D 9 |  | 4 |
| DrSSP107 | PBP27484 PBP15419 | 79.6% | MBM_03589# |  | D | L 1; D 2 |  | 4  4 |
| DrSSP108 | PBP23981 |  |  | B2J93_8640 | L | L 10 | Cutinase | 5 |
| DrSSP109 | PBP16686 |  |  | B2J93_5046# | L | L 10 | Cutinase | 6 |
| DrSSP110 | PBP24136 |  |  | B2J93_7010# | L | L 10 | ML domain | 4 |
| DrSSP111 | PBP26211 PBP21612 | 85.7% |  | B2J93_3654# | L | L 10 | Ser-Thr-rich glycosyl-phosphatidyl-inositol-anchored membrane family | 0  0 |
| DrSSP112 | PBP28594 PBP26582 | 91% |  | B2J93_4467 | L | L 9; S 1 | CFEM domain | 8  8 |
| DrSSP113 | PBP16803 |  |  | B2J93_2401 | L | L 8 |  | 4 |
| DrSSP114 | PBP18281 |  |  | B2J93_1651# | L | L 7; D 3 |  | 5 |
| DrSSP115 | PBP28021 |  |  | B2J93_3586# | S | L 5; S 2; E 3 | Necrosis inducing protein (NPP1) | 5 |
| DrSSP116 | PBP16002 |  |  | B2J93_5210# | L | L4 |  | 4 |
| DrSSP117 | PBP24251 PBP23018 | 56.6% |  | B2J93_3797# | L | L 4; D 3; S 3 | Pectate lyase | 11  8 |
| DrSSP118 | PBP23090 PBP20819 | 93.8% |  | B2J93_1408# | L | L 3 |  | 8  8 |
| DrSSP119 | PBP24975 PBP19294 | 88.7% |  | B2J93_1803# | L | L 1; S 1; F 1; Fis 1; animal 3; Fis 2; Slime mold 1 | Thioredoxin | 2  3 |
| DrSSP120 | PBP17338 |  |  | B2J93_9154 | S | L 1; S 2; |  | 8 |
| DrSSP121 | PBP28136 |  |  | B2J93_2874# | E | E 3; D 6; S 1 |  | 1 |
| DrSSP122 | PBP21014 |  |  | B2J93_9154 | S | S 2; L 2 |  | 8 |
| DrSSP123 | PBP28128 |  |  | B2J93_2866# | Unique | Unique |  | 0 |
| DrSSP124 | PBP21015 |  |  | B2J93_9154 | Unique | Unique |  | 6 |
| DrSSP125 | PBP19667 |  |  | B2J93_8846# | Unique | Unique |  | 6 |
| DrSSP126 | PBP26210 |  |  | B2J93_3653# | Unique | Unique |  | 6 |
| DrSSP127 | PBP25383 |  |  | B2J93_9600# | Unique | Unique |  | 11 |
| DrSSP128 | PBP18374 PBP15843 | 80.1% |  | B2J93_5049# | Unique | Unique |  | 2  2 |
| DrSSP129 | PBP17358 |  |  | B2J93_4738# | Unique | Unique |  | 7 |
| DrSSP130 | PBP28752 |  |  | B2J93_7959# | Unique | Unique |  | 7 |
| DrSSP131 | PBP15722 PBP15509 | 77.6% |  | B2J93_8548# | Unique | Unique |  | 8  8 |
| DrSSP132 | PBP24321 |  |  | B2J93_831 | Unique | Unique |  | 2 |
| DrSSP133 | PBP25976*  PBP26157* | 92.3% |  |  | L | L 2; D 7; M 1 | Hydrophobic surface binding protein A | 0  0 |
| DrSSP134 | PBP25574* |  |  |  | S | S 1 |  | 8 |
| DrSSP135 | PBP24819* PBP22322* | 83.9% |  |  | Unique | Unique |  | 2  2 |
| DrSSP136 | PBP26514 |  |  |  | Unique | Unique |  | 7 |
| DrSSP137 | PBP25256 PBP19996 | 82.6% |  |  | Unique | Unique |  | 6  7 |
| DrSSP138 | PBP22772* |  |  |  | Unique | Unique |  | 8 |
| DrSSP139 | PBP18380* PBP15695* | 84.4% |  |  | Unique | Unique |  | 0  0 |
| DrSSP140 | PBP23707* PBP22028* | 76.3% |  |  | Unique | Unique |  | 1  2 |
| DrSSP141 | PBP21611* |  |  |  | Unique | Unique |  | 6 |
| DrSSP142 | PBP25790* PBP22897* | 72.7% |  |  | Unique | Unique |  | 0  0 |
| DrSSP143 | PBP26102* |  |  |  | Unique | Unique |  | 5 |
| DrSSP144 | PBP26100* |  |  |  | Unique | Unique |  | 4 |
| DrSSP145 | PBP15409* |  |  |  | Unique | Unique |  | 9 |
| DrSSP146 | PBP27651* PBP20056* | 75.9% |  |  | Unique | Unique |  | 8  6 |
| DrSSP147 | PBP15414* |  |  |  | Unique | Unique |  | 4 |
| DrSSP148 | PBP15631* |  |  |  | Unique | Unique |  | 8 |
| DrSSP149 | PBP16246* |  |  |  | Unique | Unique |  | 4 |
| DrSSP150 | PBP16896* |  |  |  | Unique | Unique |  | 8 |
| DrSSP151 | PBP17092* |  |  |  | Unique | Unique |  | 9 |
| DrSSP152 | PBP17098* |  |  |  | Unique | Unique |  | 8 |
| DrSSP153 | PBP17271* |  |  |  | Unique | Unique |  | 9 |
| DrSSP154 | PBP17272* |  |  |  | Unique | Unique |  | 9 |
| DrSSP155 | PBP17279* |  |  |  | Unique | Unique |  | 8 |
| DrSSP156 | PBP17280* |  |  |  | Unique | Unique |  | 8 |
| DrSSP157 | PBP17281* |  |  |  | Unique | Unique |  | 8 |
| DrSSP158 | PBP17409* |  |  |  | Unique | Unique |  | 8 |
| DrSSP159 | PBP17604* |  |  |  | Unique | Unique |  | 5 |
| DrSSP160 | PBP17996* |  |  |  | Unique | Unique |  | 8 |
| DrSSP161 | PBP18181* |  |  |  | Unique | Unique |  | 8 |
| DrSSP162 | PBP18274* |  |  |  | Unique | Unique |  | 6 |
| DrSSP163 | PBP18980* |  |  |  | Unique | Unique |  | 1 |
| DrSSP164 | PBP19036* |  |  |  | Unique | Unique |  | 1 |
| DrSSP165 | PBP19265* |  |  |  | Unique | Unique |  | 6 |
| DrSSP166 | PBP19429* |  |  |  | Unique | Unique |  | 9 |
| DrSSP167 | PBP19476* |  |  |  | Unique | Unique |  | 0 |
| DrSSP168 | PBP19602* |  |  |  | Unique | Unique |  | 5 |
| DrSSP169 | PBP19670* |  |  |  | Unique | Unique |  | 8 |
| DrSSP170 | PBP19812* |  |  |  | Unique | Unique |  | 8 |
| DrSSP171 | PBP20053* |  |  |  | Unique | Unique |  | 7 |
| DrSSP172 | PBP20212* |  |  |  | Unique | Unique |  | 7 |
| DrSSP173 | PBP20215* |  |  |  | Unique | Unique |  | 7 |
| DrSSP174 | PBP20657* |  |  |  | Unique | Unique |  | 8 |
| DrSSP175 | PBP20723* |  |  |  | Unique | Unique |  | 8 |
| DrSSP176 | PBP20856* |  |  |  | Unique | Unique |  | 6 |
| DrSSP177 | PBP21257* |  |  |  | Unique | Unique |  | 8 |
| DrSSP178 | PBP21689* |  |  |  | Unique | Unique |  | 11 |
| DrSSP179 | PBP22133* |  |  |  | Unique | Unique |  | 7 |
| DrSSP180 | PBP22156* |  |  |  | Unique | Unique |  | 5 |
| DrSSP181 | PBP22457* |  |  |  | Unique | Unique |  | 8 |
| DrSSP182 | PBP22630* |  |  |  | Unique | Unique |  | 8 |
| DrSSP183 | PBP22734* |  |  |  | Unique | Unique |  | 5 |
| DrSSP184 | PBP22877* |  |  |  | Unique | Unique |  | 8 |
| DrSSP185 | PBP22933* |  |  |  | Unique | Unique |  | 7 |
| DrSSP186 | PBP23030* |  |  |  | Unique | Unique |  | 7 |
| DrSSP187 | PBP23100* |  |  |  | Unique | Unique |  | 6 |
| DrSSP188 | PBP23351* |  |  |  | Unique | Unique |  | 8 |
| DrSSP189 | PBP23585* |  |  |  | Unique | Unique |  | 8 |
| DrSSP190 | PBP23772* |  |  |  | Unique | Unique |  | 4 |
| DrSSP191 | PBP23822* |  |  |  | Unique | Unique |  | 8 |
| DrSSP192 | PBP23831* |  |  |  | Unique | Unique |  | 4 |
| DrSSP193 | PBP23856* |  |  |  | Unique | Unique |  | 8 |
| DrSSP194 | PBP24012* |  |  |  | Unique | Unique |  | 7 |
| DrSSP195 | PBP24027* |  |  |  | Unique | Unique |  | 8 |
| DrSSP196 | PBP24457* |  |  |  | Unique | Unique |  | 5 |
| DrSSP197 | PBP24594* |  |  |  | Unique | Unique |  | 9 |
| DrSSP198 | PBP24818* |  |  |  | Unique | Unique |  | 2 |
| DrSSP199 | PBP24939* |  |  |  | Unique | Unique |  | 0 |
| DrSSP200 | PBP25292* |  |  |  | Unique | Unique |  | 6 |
| DrSSP201 | PBP25379* |  |  |  | Unique | Unique |  | 6 |
| DrSSP202 | PBP25549* |  |  |  | Unique | Unique |  | 8 |
| DrSSP203 | PBP25562* |  |  |  | Unique | Unique |  | 8 |
| DrSSP204 | PBP25592* |  |  |  | Unique | Unique |  | 10 |
| DrSSP205 | PBP25916* |  |  |  | Unique | Unique |  | 9 |
| DrSSP206 | PBP25989* |  |  |  | Unique | Unique |  | 5 |
| DrSSP207 | PBP26159* |  |  |  | Unique | Unique |  | 6 |
| DrSSP208 | PBP26196* |  |  |  | Unique | Unique |  | 3 |
| DrSSP209 | PBP26328* |  |  |  | Unique | Unique |  | 8 |
| DrSSP210 | PBP26571* |  |  |  | Unique | Unique |  | 8 |
| DrSSP211 | PBP26576* |  |  |  | Unique | Unique |  | 6 |
| DrSSP212 | PBP26718* |  |  |  | Unique | Unique |  | 6 |
| DrSSP213 | PBP26772* |  |  |  | Unique | Unique |  | 7 |
| DrSSP214 | PBP27020* |  |  |  | Unique | Unique |  | 6 |
| DrSSP215 | PBP27141* |  |  |  | Unique | Unique |  | 8 |
| DrSSP216 | PBP27488* |  |  |  | Unique | Unique |  | 8 |
| DrSSP217 | PBP27559* |  |  |  | Unique | Unique |  | 5 |
| DrSSP218 | PBP28033* |  |  |  | Unique | Unique |  | 8 |
| DrSSP219 | PBP28065* |  |  |  | Unique | Unique |  | 4 |
| DrSSP220 | PBP28116* |  |  |  | Unique | Unique |  | 8 |
| DrSSP221 | PBP28380* |  |  |  | Unique | Unique |  | 5 |
| DrSSP222 | PBP28429* |  |  |  | Unique | Unique |  | 9 |
| DrSSP223 | PBP28465* |  |  |  | Unique | Unique |  | 8 |
| DrSSP224 | PBP28590* |  |  |  | Unique | Unique |  | 5 |
| DrSSP225 | PBP28615* |  |  |  | Unique | Unique |  | 5 |
| DrSSP226 | PBP28688* |  |  |  | Unique | Unique |  | 5 |
| DrSSP227 | PBP29014* |  |  |  | Unique | Unique |  | 5 |

a The overall nucleotide identities by alignments of protein pairs corresponding genomic DNA sequences .”

b The hits from one genus were counted only once. E, Eurotiomycetes; S, Sordariomycetes; L, Leotiomycetes; D, Dothideomycetes; X, Xylonomycetes; P, Pezizomycotina incertae sedis, C, Lecanoromycetes, O, Orbiliomycetes, A, Saccharomycetes; F, Schizosaccharomycetes; G, Basidiobolomycetes; B, Basidiomycota; Fis, Fungi incertae sedis; Slime mold, Amoebozoa Eumycetozoa; Animal, Metazoa; Plant, Viridiplantae magnoliopsida

Yellow grid, orthologs confirmed by the best-reciprocal-hit Blast

c Protein domain was predicted by Pfam (<http://pfam.xfam.org/>) and blastp in NCBI NR database.

*Species-specific SSPs

# SSPs in *M. coronaria* and *M. brunnea*.
